# The Oral Microbiome and Systemic Health: Current Insights into the Mouth–Body Connection

**DOI:** 10.3390/life16020294

**Published:** 2026-02-09

**Authors:** Ana Glavina, Dora Martić, Marija Ana Perko, Dora Mešin Delić, Antonija Tadin, Stjepanka Lešić, Daniela Šupe-Domić

**Affiliations:** 1 Subdivision of Dental Medicine, Division of Maxillofacial Surgery, University Hospital of Split, 21000 Split, Croatia; atadin@mefst.hr; 2 Department of Oral Medicine, Study of Dental Medicine, School of Medicine, University of Split, 21000 Split, Croatia; doramartic20@gmail.com (D.M.); marijaperko99@gmail.com (M.A.P.); d.mesin.st@gmail.com (D.M.D.); 3 Department of Restorative Dental Medicine and Endodontics, Study of Dental Medicine, School of Medicine, University of Split, 21000 Split, Croatia; 4 Department of Dental Medicine, Faculty of Dental Medicine and Health Osijek, Josip Juraj Strossmayer University of Osijek, 31000 Osijek, Croatia; slesic@fdmz.hr; 5 Department of Medical Laboratory Diagnostics, University Hospital of Split, 21000 Split, Croatia; 6 Department of Health Studies, University of Split, 21000 Split, Croatia

**Keywords:** oral microbiome, systemic health, dysbiosis, inflammation, narrative review

## Abstract

The oral cavity contains a complex and dynamic microbial ecosystem that plays a central role in maintaining both local and systemic homeostasis. Emerging evidence indicates that disturbances in oral microbial communities-including genetic and functional diversity within species-are associated not only with oral diseases but may also contribute to the development and progression of systemic diseases. This narrative review summarises the current state of knowledge on bidirectional interactions between oral microbial communities and major organ systems. A comprehensive search of PubMed/MEDLINE, Web of Science, Scopus, and Cochrane databases was conducted for studies published between 2019 and 2025, prioritising systematic reviews, meta-analyses, and high-quality mechanistic studies. Ultimately, 40 articles were included in the narrative synthesis. The results provide clear evidence of an association between oral dysbiosis and cardiovascular disease (CVD), type 2 diabetes mellitus (T2DM), chronic respiratory infections, and adverse pregnancy outcomes (APOs). Recent data also suggest links with neurodegenerative disorders, chronic kidney disease (CKD), autoimmune diseases, and cancer. Proposed mechanisms include transient or persistent bacteraemia, systemic inflammation caused by microbial metabolites and endotoxins, disruption of immune homeostasis, molecular mimicry, and modulation of host metabolic pathways. Despite growing evidence linking oral microbial communities to systemic health, most findings are based on observational studies, and causal relationships remain to be established through longitudinal and interventional research. Understanding the connection between the mouth and the body highlights the potential for targeting oral microbial activity, virulence factors, and host inflammatory responses in disease prevention and treatment.

## 1. Introduction

The human oral cavity is one of the most densely colonised microbial habitats in the body, harbouring more than 700 bacterial taxa, as well as archaea, fungi, protozoa, and viruses [1,2,3]. From birth, the oral microbiome develops through a sequence of microbial communities shaped by genetics, immune maturation, nutrition, hormonal status, saliva composition, oral hygiene habits and environmental influences [2,3,4]. Under physiological conditions, this ecosystem reaches a relatively stable equilibrium (eubiosis), maintained by colonisation resistance, ecological resilience and constant interactions between host and microbe [3,4,5].

In eubiosis, commensal oral microorganisms strengthen mucosal barriers, regulate epithelial turnover, stimulate the immune response, and protect against colonisation by exogenous pathogens [4,5,6]. They also help maintain balanced inflammatory tone and promote immune tolerance [3,4]. Beyond local effects, the oral microbiota plays a pivotal role in shaping systemic immune responses. Pathogen-associated molecular patterns (PAMPs) from oral bacteria activate Toll-like receptors (TLRs) and other pattern recognition receptors, leading to the production of cytokines such as interleukin 1 beta (IL-1β), interleukin 6 (IL-6), and tumour necrosis factor alpha (TNF-α). These immune mediators regulate local periodontal inflammation and exert systemic effects, linking oral dysbiosis with distant immune dysregulation and inflammatory diseases [3,4,7]. However, this balance is fragile and can be disrupted by poor oral hygiene, highly processed foods, systemic diseases, immunosuppression, antibiotic exposure, hormonal fluctuations, or tobacco use [6,8]. This disorder, known as oral dysbiosis, is characterised by reduced microbial diversity, excessive growth of pathobionts, and altered metabolic activity, and is now considered one of the main causes of common oral diseases such as caries, periodontitis, and peri-implantitis [4,5,7].

Accumulating evidence indicates that the effects of oral dysbiosis extend beyond the oral cavity [4,7,9,10]. Oral microorganisms and their products can enter the systemic circulation through ulcerated periodontal pockets, microtrauma during mastication, or invasive dental procedures, resulting in transient bacteraemia, low-grade systemic inflammation, and immune activation [11]. This has led to the concept of the “mouth–body connection”, which proposes a potential association between oral dysbiosis and the onset or progression of diseases in distant organ systems, although causality has not been firmly established [7,9,10]. Mechanistically, oral microorganisms can exert systemic effects not only via direct haematogenous dissemination but also through the release of outer membrane vesicles, microbial metabolites such as short-chain fatty acids (SCFAs), and molecular mimicry, all of which modulate distant tissue responses and immune pathways [7,9,10]. In recent years, numerous systematic reviews and meta-analyses have reinforced this concept [4,7,12,13,14,15,16,17,18].

Tobacco use, both smoked and smokeless, significantly alters the composition and diversity of the oral microbiome, promotes dysbiosis, and contributes to systemic inflammation [19]. Nicotine and other toxic components of tobacco disrupt the balance between commensal and pathogenic species, enhance the virulence of periodontopathogens, and modulate host immune responses. These changes may exacerbate local periodontal disease and increase susceptibility to systemic disease, highlighting the importance of tobacco cessation as part of oral and systemic health strategies [8].

Taken together, these findings support the view that the oral microbiome is not a passive local community but an active regulator of systemic health [7,9,10]. Dysbiosis may contribute to disease in distant organs through haematogenous dissemination of pathogens, low-grade systemic inflammation, immune dysregulation, molecular mimicry, and cross-talk with other microbial communities [7,9,10]. This microbial imbalance has important diagnostic and therapeutic implications. Salivary microbiome profiling and inflammatory markers are being explored as non-invasive biomarkers for early disease detection and risk prediction [20]. Strategies to restore microbial balance, including targeted antimicrobials, probiotics, prebiotics, synbiotics, and microbiome transplantation, are under investigation [6].

Nevertheless, major gaps remain. The causal direction between oral dysbiosis and systemic diseases is still unclear, and methodological heterogeneity across studies limits comparability and reproducibility [21]. The prognostic value of salivary biomarkers is uncertain, and it is unknown whether modulating the oral microbiome can meaningfully alter the course of systemic diseases [10,20,21]. Future research should prioritise standardised diagnostic protocols, multi-omics integration, and well-designed longitudinal studies to clarify these relationships [10,21].

This narrative review aims to synthesise current evidence (2019–2025) on the links between oral microbiome dysbiosis and systemic health, with emphasis on high-level evidence from systematic reviews, meta-analyses, and key mechanistic studies. By mapping the impact of oral dysbiosis on major organ systems and elucidating underlying mechanisms, this review seeks to highlight the diagnostic, prognostic, and therapeutic potential of targeting the oral microbiome to improve systemic health outcomes.

## 2. Materials and Methods

This narrative review focused on secondary analyses of human studies, including systematic reviews and meta-analyses, to prioritise clinically meaningful associations between the oral microbiome and systemic health. The review was conducted in accordance with the Scale for the Assessment of Narrative Review Articles (SANRA) guidelines to ensure methodological rigour, transparency, and reproducibility (Table S1). A comprehensive literature search was conducted between September and November 2025 using the PubMed (MEDLINE), Web of Science, Scopus, and Cochrane databases. The search strategy included a combination of Medical Subject Headings (MeSH) and free-text keywords such as “oral microbiome”, “oral dysbiosis”, “periodontitis”, “systemic diseases”, “neurodegenerative diseases”, “cardiovascular disease”, “infective endocarditis”, “respiratory disease”, “lung cancer”, “colorectal cancer”, “fatty liver”, “type 2 diabetes mellitus”, “gestational diabetes”, “adverse pregnancy outcomes”, “chronic kidney disease”, “autoimmune diseases”, “rheumatoid arthritis”, “Sjögren’s disease”, “oral squamous cell carcinoma”, “head and neck cancer”, “meta-analysis”, and “systematic review”. To ensure comprehensive and system-specific coverage, additional targeted keywords were used for each physiological domain, in accordance with the SANRA criteria for transparent narrative reviews.

For the neurological system, searches included “Alzheimer’s disease”, “Parkinson’s disease”, “neuroinflammation”, “cognition”, and “depression”. For the cardiovascular system, terms such as “atherosclerosis”, “endothelial dysfunction”, “nitric oxide”, “hypertension”, and “microbial translocation” were used. For the respiratory system, additional filters included “pneumonia”, “chronic obstructive pulmonary disease”, “oral–lung axis”, and “COVID-19”. For the gastrointestinal (GI) and hepatic domains, specific terms included “oral–gut axis”, “inflammatory bowel disease”, “colorectal cancer”, and “non-alcoholic fatty liver disease”. For the endocrine and metabolic systems, the search included “insulin resistance”, “gestational diabetes”, and “periodontitis-associated pregnancy outcomes”. For the renal, autoimmune, and oncological domains, additional terms such as “chronic kidney disease”, “immune dysregulation”, “rheumatoid arthritis”, “Sjögren’s disease”, and “microbiome biomarkers” were included. A snowballing approach was used by manually screening the reference lists of all included articles to identify additional relevant systematic reviews and meta-analyses. Boolean operators (AND, OR) were used to increase the sensitivity and specificity of the search, and the reference lists of all included papers were manually screened to identify further relevant studies.

The eligibility criteria were limited to systematic reviews, umbrella reviews, and meta-analyses published in peer-reviewed journals between January 2019 and July 2025, and written in English. Studies were included if they examined associations between the oral microbiome or oral dysbiosis and systemic diseases affecting any organ system. Exclusion criteria included narrative reviews without a systematic methodology, case reports, editorials, commentaries, conference abstracts, and studies based solely on in vitro or animal models. Although the primary focus was on high-level evidence from the past five years, a small number of older mechanistic or seminal studies were deliberately included due to their foundational contribution to understanding key pathophysiological mechanisms linking oral dysbiosis to systemic disease. The inclusion of these older mechanistic or seminal studies was based on the authors’ subjective judgement rather than predefined criteria, which inherently carries a risk of selection bias. All records were imported into reference management software and duplicates were removed. Two independent authors (A.G. and A.T.) screened titles and abstracts for relevance, resolving disagreements through discussion until consensus was reached. Full-text articles meeting the inclusion criteria were then reviewed in detail. Data from each eligible study were extracted according to a structured protocol, capturing information on the type of review, the number and type of primary studies included, the systemic conditions examined, main findings, reported effect estimates, and proposed biological mechanisms. As this review is narrative in nature, it is inherently limited by potential selection bias and heterogeneity in the methodologies of the included reviews, which preclude quantitative synthesis and formal risk of bias assessment.

As most of the included studies were systematic reviews and meta-analyses, no new quantitative meta-analysis was conducted. Formal certainty-of-evidence assessments (e.g., Grading for Recommendations Assessment, Development and Evaluation, GRADE) were not consistently reported in the included meta-analyses. Therefore, no additional quality grading was applied in this review. Instead, the evidence was synthesised qualitatively and presented thematically by organ system, including neurological, cardiovascular, respiratory, GI/hepatic, endocrine/metabolic, renal, autoimmune, and oncological domains. Mechanistic pathways were analysed by distinguishing between those shared across multiple systemic diseases and those that are disease-specific, based on available evidence from both high-level reviews and selected original studies. Particular emphasis was placed on the strength and consistency of reported associations, the biological plausibility of proposed mechanisms, and the identification of existing knowledge gaps. As this study did not involve primary research with human participants, approval from an institutional ethics committee was not required.

## 3. Results

**Figure 1:**
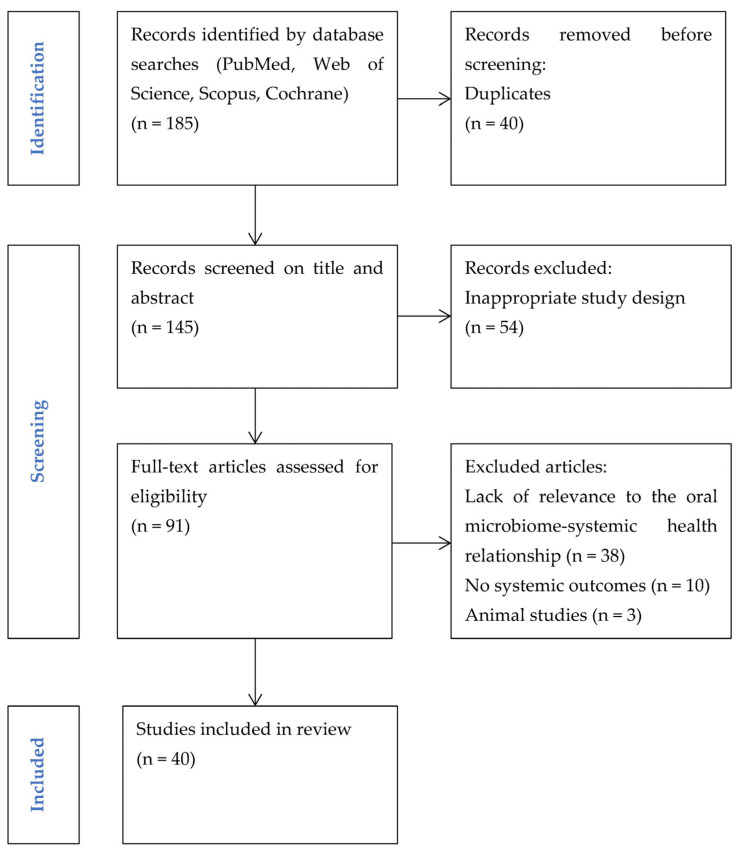
Study selection.

A total of 185 articles were identified through searches of the aforementioned electronic databases. After removing 40 duplicates, 145 articles remained and were screened by title and abstract. During screening, 54 records were excluded for not meeting the predefined inclusion criteria due to inappropriate study design. The full texts of 91 articles were assessed for eligibility. Of these, 51 articles were excluded due to lack of relevance to the oral microbiome–systemic health relationship, absence of reported systemic outcomes in human studies, or exclusive focus on animal or in vitro experimental models, which were outside the scope of this review. Finally, 40 articles were included in the narrative synthesis. A quantitative synthesis (meta-analysis) was not performed, in accordance with the aims and design of this narrative review. The selection process is illustrated in the Preferred Reporting Items for Systematic Reviews and Meta-Analyses (PRISMA) flow diagram (Figure 1). Although this work is a narrative review, selected PRISMA reporting elements, including the flow diagram, were used to enhance the transparency and reproducibility of the literature search and selection process.

### 3.1. Evidence Overview

Evidence from over 30 systematic reviews and meta-analyses, covering more than 1000 primary studies (2019–2025), demonstrates measurable systemic immunometabolic effects of oral dysbiosis across organ systems. A consistent systemic immunometabolic signature is characterised by chronic low-grade cytokinaemia (IL-1β, IL-6, TNF-α), oxidative–nitrosative imbalance, endothelial activation, and immune maladaptation. Taxonomically, dysbiosis is marked by expansion of proteolytic anaerobes (*Porphyromonas gingivalis*, *Fusobacterium nucleatum*, *Prevotella intermedia*) and depletion of nitrate-reducing commensals (*Neisseria*, *Rothia*, *Haemophilus*). These shifts have been reported to be associated with activation of TLR2/4–nuclear factor kappa B (NF-κB)–NOD-like receptor pyrin domain-containing 3 (NLRP3) signalling, increased accumulation of reactive oxygen species and reactive nitrogen species (ROS/RNS), and suppression of the nitrate–nitrite–nitric oxide (NO) axis. This leads to a chronic, low-grade systemic inflammatory state linking oral and extra-oral pathology. However, these observations are largely based on experimental or indirect evidence and require further confirmation, particularly in clinical settings.

### 3.2. Domain-Specific Evidence and Quantitative Signals

#### 3.2.1. Neurological and Neurodegenerative Disorders

Umbrella-level evidence links oral dysbiosis to dementia and cognitive decline (pooled OR ~1.7, 95% CI 1.3–2.2) [22,23,24,25,26,27,28,29,30,31,32,33,34,35]. Where reported, these associations have been accompanied by substantial heterogeneity across studies (significant I^2^), reflecting methodological and population diversity among component studies, which should temper interpretation of pooled estimates. *P. gingivalis* gingipains promote tau phosphorylation and β-amyloid aggregation via NF-κB/mitogen-activated protein kinase (MAPK) signalling. Bacterial lipopolysaccharides (LPS) and cytokines disrupt the blood–brain barrier (BBB) and activate microglia. A recent Mendelian randomisation (MR) analysis found that genetically proxied periodontitis affects brain cortical structure, impacting several cortical regions involved in neurodegenerative and neuroinflammatory processes [36]. This provides genetic evidence for a biological link between periodontal inflammation and brain morphology, a key substrate for neuroinflammation. Another comprehensive bidirectional MR study examined periodontitis and brain atrophy or cognitive impairment but did not find strong causal associations in either direction, highlighting the need for further research into genetic causal pathways between periodontitis and neurological outcomes [37]. These neuroimmune processes parallel vascular inflammatory cascades described in cardiometabolic disorders.

#### 3.2.2. Cardiovascular and Vascular–Metabolic Disease

Meta-analyses show that periodontal therapy reduces C-reactive protein (CRP) by 1–3 mg/L and IL-6 by approximately 30%, with improved flow-mediated dilation. Severe periodontitis is associated with atherosclerotic disease (RR ~1.6, 95% CI 1.2–2.0) [38,39,40,41,42,43,44]. Where reported, substantial between-study heterogeneity has been observed (e.g., I^2^ = 85% in analyses of carotid atherosclerosis), indicating variability across study designs and populations, and limiting the interpretability of pooled estimates. Bondonno et al. conducted a randomised, controlled, cross-over trial in which hypertensive adults used an antibacterial mouthwash for three days. This intervention reduced oral nitrate-to-nitrite conversion (disrupting the nitrate–nitrite–NO pathway) and was associated with a small but statistically significant increase in systolic blood pressure (~+2.3 mmHg) compared with control (water) rinsing [45]. These data define a microbiome-driven endothelial axis linking oral inflammation with vascular rigidity and hypertension.

#### 3.2.3. Respiratory and Infectious Diseases

Systematic reviews indicate that structured oral care programmes reduce pneumonia incidence in institutionalised adults by approximately 40% (RR 0.60, 95% CI 0.45–0.80) [13,19,46,47,48,49]. In meta-analyses where data were pooled (e.g., RR ~0.61 for non-ventilated adults), heterogeneity metrics were not consistently reported, reflecting methodological diversity among trials and limiting the ability to conclusively quantify I^2^ across these studies. Where heterogeneity was assessed, variability in interventions and study designs was noted, further limiting the interpretability of pooled estimates. Shared taxa (*Prevotella*, *Veillonella*, *Haemophilus*) across oral–airway ecosystems sustain airway inflammation. In Coronavirus Disease 2019 (COVID-19), enrichment of *Prevotella* and *Fusobacterium* correlates with higher IL-6 and interleukin 10 (IL-10) concentrations and more severe pulmonary involvement, implicating oral dysbiosis as a co-determinant of viral pathogenesis.

#### 3.2.4. Gastrointestinal and Hepatic Disorders

High-confidence reviews report that oral-origin taxa (*F. nucleatum*, *P. gingivalis*) are enriched in colorectal cancer (CRC) (pooled OR ~2.2, 95% CI 1.5–3.0), with mechanistic activation of β-catenin/NF-κB, DNA damage, and mitochondrial dysfunction [14,15,50,51,52,53,54,55]. Where reported in the original meta-analyses, heterogeneity indices have varied: for example, moderate between-study heterogeneity (I^2^ ≈ 39%) has been observed in pooled analyses of *F. nucleatum* abundance in CRC tissue, and high heterogeneity (I^2^~83%) has been reported for some molecular associations, reflecting variability in study populations, detection methods, and specimen types. Oral dysbiosis is linked to non-alcoholic fatty liver disease (NAFLD) via translocated microbial products that activate Kupffer cells and promote oxidative fibrogenesis. Loss of nitrate-reducers reduces hepatic NO bioavailability. Combined salivary and faecal microbial panels improve early CRC detection compared with faecal immunochemical testing.

#### 3.2.5. Endocrine and Reproductive Systems

Meta-analyses show a bidirectional association between periodontitis and type 2 diabetes mellitus (T2DM) (pooled OR ~1.5–1.8). Periodontal therapy reduces glycated haemoglobin (HbA1c) by about 0.4–0.6% [16,56]. Meta-analyses examining this bidirectional association report pooled effect estimates with moderate to high heterogeneity (e.g., summary risk ratio (SRR) ≈ 1.26 for periodontitis preceding T2DM with I^2^ ≈ 71%, and SRR ≈ 1.24 for T2DM preceding periodontitis with I^2^ ≈ 92%), reflecting variability in study populations, diagnostic criteria, and confounder adjustments. During pregnancy, *P. gingivalis* and *F. nucleatum* activate placental TLR4 and oxidative stress, increasing the risk of pre-eclampsia (RR ~1.7, 95% CI 1.2–2.4), consistent with an oral–placental–vascular triad mediated by cytokines and NO depletion [57,58]. While meta-analytic evidence linking periodontitis to pre-eclampsia has been reported, evidence of between-study heterogeneity has ranged from moderate (I^2^ ≈ 55%) to high (I^2^ ≈ 89%), reflecting variability in study designs, definitions of periodontal exposure, and populations.

#### 3.2.6. Renal and Autoimmune Diseases

Periodontitis is associated with an increased risk of chronic kidney disease (CKD) (~1.5-fold, 95% CI 1.1–2.0) [20,59,60]. Cytokine spillover and endotoxaemia exacerbate glomerular inflammation, while uraemia selects for proteolytic anaerobes. In meta-analytic evidence, this association is accompanied by moderate between-study heterogeneity (I^2^ ≈ 35%), indicating some variability between studies but overall reasonable consistency in the direction and magnitude of the effect. In contrast, reverse associations (CKD → periodontitis) exhibit high heterogeneity (I^2^ ≈ 90%), reflecting greater variability in observational designs and diagnostic criteria across studies. In autoimmunity, *P. gingivalis* peptidylarginine deiminase (PPAD) generates citrullinated neoantigens, and *Aggregatibacter actinomycetemcomitans* induces neutrophil extracellular trap formation (NETosis)—mechanisms linked to rheumatoid arthritis (RA) and Sjögren’s disease (SjD) [17,61,62].

#### 3.2.7. Oncological Disorders

Meta-analyses consistently show enrichment of *F. nucleatum* and *P. gingivalis* in oral and colorectal tumours (pooled OR ~2.3, 95% CI 1.6–3.4), with activation of NF-κB, signal transducer and activator of transcription 3 (STAT3), β-catenin signalling, and epithelial–mesenchymal transition (EMT), promoting angiogenesis, DNA damage, and immune evasion [14,15,51,52,53,54]. Where reported, between-study heterogeneity has ranged from moderate to high, such as I^2^ ≈ 60% for tumour site associations and differential tumour characteristics, although some stratified analyses show lower heterogeneity (I^2^ ≈ 0–29%) depending on outcome and study subsets. This variability reflects differences in specimen types, detection methods, and study populations. Carcinogenic metabolites (acetaldehyde, nitrosamines) further potentiate genomic instability, especially with tobacco or alcohol exposure.

### 3.3. Cross-System Mechanistic Integration

Table 1 summarises meta-analytic evidence for major organ systems, and Table 2 presents quantitative parameters.

### 3.4. Translational Implications

Although much of the evidence remains associative, longitudinal and MR data now provide early causal inference. Interventional trials show that periodontal therapy consistently lowers systemic inflammatory markers, improves endothelial and metabolic indices, and reduces the risk of preterm birth. The oral microbiome may therefore represent a modifiable factor influencing systemic homeostasis. Approaches such as targeted microbial modulation with probiotics or synbiotics, nitrate-preserving oral care, and cytokine-attenuating periodontal therapy have been proposed as potential precision health strategies to support immunometabolic balance, although further clinical validation is required. Table 2 presents a quantitative synthesis of mechanistic pathways linking oral dysbiosis to systemic inflammation, oxidative stress, metabolic endotoxaemia, immune maladaptation, oncogenesis, and vascular dysregulation across meta-analyses from 2019 to 2025.

**Figure 2:**
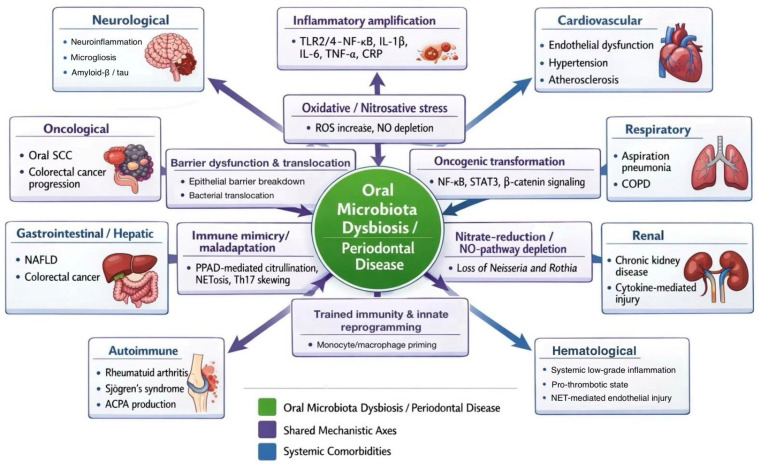
Schematic representation of local and systemic inflammatory pathways linking oral dysbiosis and periodontal disease to associated comorbidities. Abbreviations: OSCC, oral squamous cell carcinoma; NAFLD, non-alcoholic fatty liver disease; ACPA, anti-citrullinated protein antibody; TLR, Toll-like receptor; NF-κB, nuclear factor kappa B; IL-1β, interleukin-1 beta; IL-6, interleukin 6; TNF-α, tumour necrosis factor alpha; CRP, C-reactive protein; ROS, reactive oxygen species; NO, nitric oxide; PPAD, Porphyromonas gingivalis peptidylarginine deiminase; NETosis, neutrophil extracellular trap; STAT3, signal transducer and activator of transcription 3; COPD, chronic obstructive pulmonary disease.

In addition, there is a schematic explanatory figure (Figure 2), based on previously published conceptual frameworks (Table 1 and Table 2), that links oral dysbiosis and periodontal disease to systemic inflammatory comorbidities.

## 4. Discussion

### 4.1. Neurological System

#### 4.1.1. Alzheimer’s Disease (AD)

Accumulating evidence highlights the significant role of oral microbiota dysbiosis in the pathogenesis and progression of AD. Several meta-analyses and systematic reviews have consistently reported alterations in the oral microbial ecosystem among AD patients, characterised by increased abundance of *P. gingivalis*, *Treponema denticola*, and *F. nucleatum*, alongside depletion of commensal genera such as *Streptococcus* and *Actinomyces* [29,30]. This dysbiosis contributes to systemic inflammation, endothelial dysfunction, and increased BBB permeability, facilitating the translocation of bacterial virulence factors, including exosomes and endotoxins, into the central nervous system (CNS). Maitre et al. synthesised evidence linking gingipains from *P. gingivalis* to microglial activation and neurodegeneration, showing that these cysteine proteases induce tau hyperphosphorylation and amyloid-β aggregation via NF-κB and MAPK signalling cascades [24]. Chronic exposure to microbial LPS increases proinflammatory cytokine release (IL-1β, IL-6, TNF-α) and ROS, amplifying neuronal injury and synaptic dysfunction. In addition to microbial mechanisms, behavioural factors such as chronic alcohol use exacerbate dysbiosis and accelerate neurodegeneration [25]. The systematic review by Chaple-Gil et al. demonstrated a significant association between oral microbial imbalance and overall dementia risk, suggesting that oral dysbiosis could serve as an early, non-invasive biomarker of neurodegenerative susceptibility [28]. Mao et al. further proposed that chronic periodontitis triggers peripheral inflammation and immune priming, promoting β-amyloid deposition and amplifying neuroinflammation [29].

Longitudinal data support these mechanistic links: Karaduran et al. observed that periodontitis independently predicted faster cognitive decline and greater AD progression in a prospective cohort [30]. In contrast, Jockusch et al. reported that cognitive impairment leads to poorer oral hygiene and reduced use of dental services, reinforcing the concept of a bidirectional relationship between neurodegeneration and oral health [31]. Collectively, these studies support a model in which oral dysbiosis sustains systemic inflammation, promotes neuroimmune activation, and accelerates AD pathology.

#### 4.1.2. Parkinson’s Disease (PD)

Emerging evidence suggests that disturbances in the oral microbiota may contribute to the pathophysiology of PD. Murcia-Flores et al. demonstrated consistent microbial alterations in patients with PD, including an overrepresentation of *P. intermedia* and *Porphyromonas* spp., and reduced diversity of *Lactobacillus* [26]. These changes correlate with increased systemic oxidative stress, altered mucosal immunity, and α-synuclein aggregation in enteric and central neurons.

The oral–gut–brain axis has been proposed as a mechanistic framework linking microbial dysbiosis to neuroinflammation. Barrio et al. reviewed psychobiotic and immunomodulatory interactions between the gut and brain, describing how microbial metabolites such as SCFAs modulate microglial reactivity and dopaminergic function [69]. Rei et al. conceptualised the oral and nasal microbiota as “microbial conductors” orchestrating local and systemic inflammatory signals, with the potential to influence nigrostriatal neurodegeneration through TLR4 and inflammasome pathways [64]. Experimental findings by Wang et al. support these hypotheses, showing that oral microbial components can cross the mucosal barrier, activate microglia, and induce dopaminergic neuronal apoptosis via TNF-α- and IL-6-dependent mechanisms [70].

These data collectively underscore the role of the oral microbiome as both a diagnostic biomarker and a therapeutic target in PD. Interventions to restore microbial homeostasis, such as improved oral hygiene, probiotics, or targeted antimicrobial therapy, may reduce systemic inflammation and slow neurodegenerative processes.

#### 4.1.3. Neuroinflammation, Cognition and Depression

The concept of the oral–gut–brain axis extends beyond neurodegeneration to include neuroinflammatory and neuropsychiatric disorders. Chronic oral dysbiosis is increasingly recognised as a peripheral driver of neuroimmune activation. Paudel et al. demonstrated that psychological stress causes significant changes in both oral and gut microbial composition, activates the hypothalamic–pituitary–adrenal (HPA) axis, and increases systemic inflammatory mediators [32]. These findings support the emerging understanding that chronic stress promotes dysbiosis, elevates circulating LPS levels, and impairs serotonergic and dopaminergic neurotransmission. Adil et al. further consolidated evidence linking oral–gut microbial networks with cognitive performance, showing that microbial metabolites such as SCFAs, tryptophan derivatives, and bile acids mediate cross-talk between mucosal immunity and cortical neuronal activity [33]. Clasen et al. identified virulence signatures across the oral–gut–brain continuum that modulate both PD and cognitive decline, implicating bacterial amyloids and proinflammatory cytokines as shared pathological mediators [34].

From a psychiatric perspective, García-Rios et al. systematically reviewed the association between oral dysbiosis and depression, demonstrating consistent correlations between reduced microbial diversity, elevated systemic cytokines (IL-1β, IL-6, TNF-α), and dysregulation of neurotrophic factors such as brain-derived neurotrophic factor (BDNF) [35]. This growing body of evidence suggests that oral health may be an often overlooked but potentially modifiable factor associated with mental and cognitive well-being. Preventive strategies, including regular dental care, improved oral hygiene, and targeted modulation of the oral microbiome, have significant potential to reduce systemic inflammation and support mental and cognitive health.

### 4.2. Cardiovascular System

#### 4.2.1. Overview of the Oral–Vascular Axis

Cardiovascular diseases (CVD) remain the leading global cause of morbidity and mortality, with increasing evidence implicating oral microbiota dysbiosis as an independent and modifiable risk factor. Chronic periodontitis, gingival inflammation, and microbial imbalance have been associated with systemic immune activation, endothelial injury, and metabolic dysregulation, suggesting a potential link between local oral pathology and systemic vascular outcomes [38,39,40].

The Cochrane meta-analysis by Ye et al. found that periodontal therapy significantly reduces systemic inflammatory markers (CRP, IL-6) and improves endothelial function, highlighting a causal link between oral inflammation and vascular disease [38]. Pizziolo et al. also demonstrated that tooth loss and oral dysbiosis are associated with elevated blood pressure, arterial stiffness, and increased carotid intima–media thickness [39]. These associations persist after adjustment for traditional CVD risk factors, indicating a biological connection rather than a simple correlation. The oral–vascular axis thus forms a systemic network integrating microbial, immune, and metabolic pathways that influence vascular tone, endothelial homeostasis, and atherogenesis.

#### 4.2.2. Oral Dysbiosis and Vascular Systemic Inflammation

Oral dysbiosis promotes the chronic release of bacterial virulence factors, such as LPS, gingipains, and SCFA metabolites, into the systemic circulation. These components activate TLR2, TLR4, and NOD-like receptors (NLRs) on endothelial and immune cells, initiating NF-κB–driven cytokine cascades (IL-1β, TNF-α, IL-6). Persistent activation of these inflammatory pathways leads to vascular endothelial dysfunction, oxidative stress, and prothrombotic signalling [38,40,43].

Akhi et al. identified increased antibody titres against *P. gingivalis* and *Tannerella forsythia* in patients with both periodontitis and atherosclerosis, implicating molecular mimicry between bacterial and host heat shock proteins (HSP60/65) as a mechanism for autoimmune endothelial injury [40]. Furthermore, *P. gingivalis* outer membrane vesicles have been shown to penetrate endothelial monolayers and disrupt tight junctions, increasing vascular permeability and promoting monocyte adhesion [40]. These findings identify oral dysbiosis as both a source of systemic inflammatory burden and a driver of vascular immune dysregulation.

#### 4.2.3. Microbial Translocation and Atherosclerosis

Wu et al. demonstrated that patients with carotid atherosclerosis have distinct salivary microbial and metabolomic profiles, characterised by increased abundance of *Prevotella*, *Veillonella*, and *Campylobacter*, and reduced levels of nitrate-reducing *Neisseria* and *Rothia*. This shift correlates with elevated serum CRP and increased carotid plaque burden [41]. Mechanistically, bacterial components can induce endothelial activation and expression of adhesion molecules (intercellular adhesion molecule-1 (ICAM-1), vascular cell adhesion molecule-1 (VCAM-1)), facilitating monocyte recruitment and foam cell formation, while SCFAs derived from dysbiotic biofilms alter lipid metabolism and macrophage polarisation, contributing to plaque instability. Overall, alterations in the salivary microbiome support a link between oral dysbiosis and atherogenesis [41].

#### 4.2.4. Nitric Oxide Pathways and Hypertension

The discovery of the oral–nitrate–NO axis has transformed our understanding of microbial contributions to vascular physiology. Commensal nitrate-reducing bacteria such as *Neisseria*, *Haemophilus*, and *Veillonella* convert dietary nitrate (NO_3_^−^) into bioactive nitrite (NO_2_^−^), which serves as a substrate for systemic NO production, a key vasodilator and regulator of endothelial tone [43]. Murugesan and Al Khodor showed that reduced microbial diversity and depletion of nitrate-reducing taxa correlate with elevated blood pressure in hypertensive subjects, indicating that oral microbial ecology modulates vascular homeostasis [42]. Disruption of this nitrate–NO pathway through excessive use of antiseptic mouthwashes or antibiotics suppresses beneficial commensals, decreases NO bioavailability, and promotes vasoconstriction [42,43]. Morou-Bermúdez et al. described a mechanism in which reduced nitrate reductase activity in oral bacteria leads to decreased NO synthesis, accumulation of ROS, and increased levels of asymmetric dimethylarginine (ADMA), an endogenous nitric oxide synthase (NOS) inhibitor [43]. These findings highlight a microbiome-dependent regulatory system for blood pressure and vascular tone, suggesting that maintaining oral microbial balance may play an important role in supporting cardiovascular homeostasis. They underscore the potential of personalised preventive and point-of-care strategies, such as individualised oral hygiene programmes, microbiome monitoring, and targeted interventions to maintain oral microbial balance and support cardiovascular homeostasis.

#### 4.2.5. Oral Microbiota and Infective Endocarditis (IE)

Beyond chronic vascular disease, the oral microbiome directly contributes to IE, a potentially fatal inflammation of the heart valves. Gomes et al. [44] used next-generation sequencing (NGS) to demonstrate microbial overlap between endodontic–periodontal infections and IE isolates, identifying *Streptococcus sanguinis*, *F. nucleatum*, and *P. intermedia* as common pathogens. Bacteraemia resulting from routine oral activities such as chewing or brushing is typically transient in healthy individuals but can cause endothelial colonisation in patients with valvular lesions or prosthetic devices. Subsequent fibrin–platelet aggregation around the bacteria forms vegetations, perpetuating inflammation and embolic complications. Molecular analyses have detected *P. gingivalis* and *T. denticola* DNA in cardiac tissues and atherosclerotic plaques, supporting the haematogenous dissemination route [44]. These findings support the inclusion of oral health screening and targeted antibiotic prophylaxis in cardiovascular prevention protocols, particularly for high-risk populations.

#### 4.2.6. Therapeutic Perspectives

Periodontal and microbiome-targeted therapies significantly reduce systemic inflammation and improve vascular function [38,39]. Salivary microbiome analysis and nitrate reductase activity profiling may soon serve as non-invasive biomarkers for early detection of cardiovascular risk [41,42]. Emerging research supports the integration of dental and cardiovascular disciplines within a unified precision medicine framework, in which oral microbial modulation serves as an adjunctive strategy for preventing atherosclerosis, hypertension, and endothelial disease.

### 4.3. Respiratory System

#### 4.3.1. Overview of the Oral–Lung Axis

The oral cavity and lower respiratory tract form a continuous ecological and immunological interface known as the oral–lung axis. Salivary flow, microaspiration, and mucosal aerosolisation enable bidirectional microbial and metabolic communication between these two compartments [13,19,49]. In health, this axis maintains mucosal immune tolerance and supports airway epithelial homeostasis through microbial metabolites and local cytokine regulation. However, dysbiosis of the oral microbiota, characterised by overgrowth of anaerobic and proteolytic taxa such as *P. gingivalis*, *F. nucleatum*, and *P. intermedia*, can alter host immune responses and promote inflammatory propagation to distal airways [13,49]. Saliva serves as a reservoir and transport medium for microorganisms that can colonise the respiratory tract during sleep or in individuals with impaired swallowing reflexes. The presence of shared microbial signatures across oral, airway, and pulmonary niches supports the concept of an oral–airway microbial continuum [19,49].

#### 4.3.2. Aspiration Pneumonia

Aspiration pneumonia remains a leading cause of morbidity and mortality among elderly and institutionalised patients. In frail or institutionalised older adults, impaired swallowing and poor oral hygiene increase colonisation by anaerobic pathobionts and the risk of aspiration pneumonia [13,48]. Meta-analytical data indicate that structured oral hygiene interventions and regular professional cleaning significantly reduce pneumonia incidence in nursing home residents [48]. Mechanistically, bacterial LPS and proteolytic enzymes from oral biofilms activate TLR2 and TLR4 on respiratory epithelial cells, initiating NF-κB–driven cytokine release (IL-1β, IL-6, TNF-α), neutrophil recruitment, and oxidative stress [13,48]. This leads to mucociliary dysfunction, epithelial damage, and an exaggerated inflammatory response that predisposes individuals to secondary bacterial infections and recurrent pneumonia. Maintaining oral microbial balance is therefore a crucial preventive measure against aspiration-related pulmonary disease.

#### 4.3.3. Chronic Obstructive Pulmonary Disease (COPD)

The oral microbiota plays a crucial role in the chronic inflammation observed in COPD. A recent systematic review and meta-analysis identified overlapping dysbiotic patterns in the oral, airway, and intestinal microbiomes of COPD patients, specifically the enrichment of *Haemophilus*, *Prevotella*, and *Veillonella*, and the depletion of *Neisseria* and *Streptococcus* [49]. These taxa are associated with proinflammatory cytokine profiles and oxidative stress in both oral and airway mucosa. Oral dysbiosis contributes to systemic inflammation through bacteraemia, endotoxin release, and shared immune pathways. Swallowed and aspirated dysbiotic biofilms continuously seed the respiratory tract, sustaining inflammation by activating pattern recognition receptors and producing ROS [13,49]. Although DNA from oral bacteria is frequently detected in bronchoalveolar lavage fluid, evidence for viable oral bacteria in the lungs remains limited [71]. In this context, periodontal assessment and control of oral inflammation are biologically justified adjuncts to COPD care to reduce systemic inflammatory burden [13,55]. Restoration of oral eubiosis and reduction in biofilm load through periodontal therapy may therefore mitigate airway inflammation and improve respiratory outcomes.

#### 4.3.4. Viral Infections and COVID-19

Recent evidence highlights the modulatory role of the oral microbiota in viral respiratory infections, particularly COVID-19. Meta-analyses have reported significant changes in oral microbial composition among COVID-19 patients, including reduced alpha diversity and an overrepresentation of opportunistic taxa such as *Prevotella*, *Veillonella*, and *Fusobacterium* [19,46,49]. These dysbiotic communities are associated with elevated systemic cytokines (IL-6, IL-10) and severe pulmonary involvement. Dysbiotic oral and oropharyngeal communities can modulate epithelial angiotensin-converting enzyme 2 (ACE2) expression, dampen type I/III interferon responses, and degrade barrier integrity, providing plausible routes for worse viral outcomes [19,46,49]. A systematic review and meta-analysis of oral health conditions in COVID-19 patients found that periodontal symptoms were associated with increased COVID-19 severity, although study quality was generally low and causality could not be established [72]. Despite emerging associations between oral dysbiosis and COVID-19 severity, the evidence remains preliminary, with cross-sectional designs predominating and substantial heterogeneity among studies. Oral and oropharyngeal microbiome alterations thus represent both a biomarker and a potential cofactor in COVID-19 pathogenesis. In addition, oral biofilms may serve as reservoirs for secondary bacterial superinfections that worsen COVID-19 progression and respiratory failure [19,46,49]. Maintaining oral health and microbial balance may therefore contribute to improved clinical outcomes during viral respiratory infections.

#### 4.3.5. Lung Cancer

Epidemiological and mechanistic data increasingly link chronic oral inflammation and microbial dysbiosis to a higher risk of lung cancer. A systematic review found that patients with periodontal disease and specific oral microbial signatures have a significantly higher incidence of lung cancer [13,19]. Potential mechanisms include systemic dissemination of bacterial virulence factors, induction of chronic oxidative stress, and production of carcinogenic metabolites, particularly acetaldehyde and nitrosamines generated by anaerobic biofilms [13,19]. Persistent systemic inflammation originating from periodontal tissues may also create a pro-oncogenic microenvironment in pulmonary tissue through sustained activation of the NF-κB and STAT3 signalling pathways. Activation of the inflammasome, particularly NLRP3, by oral bacteria plays a central role in driving local oral inflammation and may contribute to systemic inflammatory signalling, further promoting a pro-oncogenic microenvironment in distant tissues such as the lungs. These findings suggest that oral dysbiosis contributes not only to respiratory infection but also to tumourigenic processes in the lung.

#### 4.3.6. Shared Inflammatory and Metabolic Pathways

The mechanistic link between oral dysbiosis and respiratory disease involves the convergence of microbial translocation, immune dysregulation, and metabolic imbalance. Continuous microaspiration allows oral microorganisms and their metabolites to enter the lower airways, activating epithelial TLR2/4 and nucleotide-binding oligomerisation domain (NOD) 1/2 receptors [13,49]. This triggers NF-κB activation, cytokine release, and recruitment of neutrophils and macrophages, promoting a self-perpetuating inflammatory cycle [13,49].

ROS produced by host cells and bacterial metabolism further damage epithelial barriers, while proteolytic enzymes degrade junctional complexes and increase permeability. Metabolically, depletion of nitrate-reducing taxa such as *Neisseria* and *Rothia* impairs nitrate-to-nitrite conversion, reducing NO availability and compromising bronchodilatory and antimicrobial defences [42,43]. This nitrate–NO pathway, which is critical for airway smooth muscle tone and host defence, is often suppressed in dysbiosis or by excessive use of antiseptics, contributing to airway hyperreactivity and chronic inflammation [42,43,49].

#### 4.3.7. Oral–Airway Biofilm Ecology and Microbial Continuum

Advanced sequencing studies confirm that lower-airway microbiota largely reflect the composition of oral biofilms, supporting the model of a continuous microbial ecosystem [19,49]. In dysbiosis, however, aspiration of anaerobic oral biofilms leads to the establishment of persistent polymicrobial communities in the bronchioles and alveoli. These biofilms exhibit metabolic cooperation and quorum sensing, which enhance virulence, resist clearance, and amplify inflammation [49]. Over time, this contributes to chronic airway remodelling, reduced mucociliary function, and increased susceptibility to secondary infection. Importantly, intervention studies demonstrate that improving oral hygiene and reducing periodontal inflammation significantly lower respiratory infection rates and hospital admissions in high-risk populations [13,48], highlighting the translational relevance of oral care for preventing pulmonary disease. Periodontal therapy, structured oral hygiene, and maintenance of nitrate-reducing commensals may therefore represent low-risk adjunctive strategies to reduce respiratory morbidity.

### 4.4. Gastrointestinal and Hepatic System

#### 4.4.1. Overview of the Oral–Gut Axis

The oral and intestinal microbiomes form a continuous microbial and immunometabolic network known as the oral–gut axis. Each day, more than 10^8^ bacterial cells from saliva are swallowed, seeding the gastrointestinal tract and contributing to microbial cross-colonisation [9,10]. However, oral dysbiosis, characterised by the overgrowth of anaerobic, proteolytic, and pro-inflammatory taxa such as *Fusobacterium*, *Porphyromonas*, *Prevotella*, and *Peptostreptococcus*, disrupts this equilibrium and triggers gut inflammation and metabolic endotoxaemia [50,55]. Recent bidirectional MR evidence demonstrates a causal, two-way relationship between periodontitis and gut microbiome composition [56]. Periodontitis-associated dysbiosis increases systemic cytokines (IL-1β, TNF-α, IL-6) and compromises intestinal barrier integrity, while gut dysbiosis reciprocally promotes systemic inflammation and periodontal destruction.

#### 4.4.2. Oral Dysbiosis and Inflammatory Bowel Disease (IBD)

Genetic evidence linking periodontitis to changes in gut composition, including taxa relevant to butyrate balance, provides a mechanistic basis for oral–intestinal immune coupling in IBD [50]. Although the direct and consistent detection of oral taxa within IBD mucosa varies across studies, shared TLR/NOD-driven cytokine circuits and barrier disruption offer a plausible framework by which oral dysbiosis could modulate intestinal inflammatory activity [50]. Oral-derived LPS and proteases activate intestinal epithelial TLR2/4 and NOD pathways, inducing NF-κB-mediated cytokine release and compromising mucosal tight junctions. The resulting increase in gut permeability promotes systemic endotoxaemia and amplifies local immune activation. Chen et al. demonstrated that genetic predisposition to periodontitis correlates with altered abundance of butyrate-producing genera in the gut, implying that oral inflammation directly shapes intestinal microbial ecology [50]. Conversely, intestinal dysbiosis may influence oral immunity through shared Th17-driven inflammatory pathways.

#### 4.4.3. Oral Microbiota and Colorectal Carcinogenesis

Among gastrointestinal diseases, the association between oral pathogens and CRC is particularly strong. Certain oral bacteria, such as *F. nucleatum*, which colonise the oral cavity, express exotoxins (e.g., Fusobacterium adhesin A (FadA) and Fusobacterium adhesin protein 2 (Fap2)) that modulate host signalling pathways, promote inflammation, and contribute to colorectal carcinogenesis. Multiple reviews document the enrichment of oral-origin taxa, especially *F. nucleatum* and *Peptostreptococcus*, in CRC, with convergent evidence from saliva, stool, and tumour tissue [14,15]. A systematic review focused on *P. gingivalis* reports an association with CRC, supporting its inclusion in broader consortia [54]. Camañes-Gonzalvo et al. reported consistent enrichment of these taxa in CRC patients, regardless of geographic or methodological differences [14]. Navarro-Sánchez et al. further confirmed the strong correlation between *P. gingivalis* abundance and advanced CRC stages [53]. Mechanistically, these organisms modulate epithelial proliferation and apoptosis, induce DNA damage via ROS production, and promote pro-oncogenic signalling through activation of the β-catenin and NF-κB pathways.

Beyond direct microbial invasion, dysbiosis-induced metabolic alterations, such as SCFA imbalance, secondary bile acid accumulation, and increased ammonia production, create a tumour-promoting microenvironment [14,15]. A meta-analysis of gut microbiota signatures in CRC revealed characteristic depletion of beneficial *Lachnospiraceae* and *Ruminococcaceae*, alongside overrepresentation of *Fusobacterium* and *Peptostreptococcus*, highlighting the functional overlap between oral and colonic dysbiosis [14,51].

#### 4.4.4. Microbiome-Derived Biomarkers for Early CRC Detection

The diagnostic potential of oral and gut microbial signatures has attracted increasing attention. Zwezerijnen-Jiwa et al. systematically reviewed microbiome-based biomarkers for early CRC screening and identified oral taxa such as *Fusobacterium* and *Peptostreptococcus* as highly discriminative in both stool and saliva samples [51]. Integrating salivary and faecal microbiome profiling improved diagnostic accuracy beyond conventional faecal immunochemical testing, suggesting that combining oral and intestinal microbial biomarkers could enhance non-invasive cancer detection strategies [14,15]. Reitano et al. also demonstrated that oral microbial alterations precede digestive cancer diagnosis, highlighting the feasibility of salivary microbial surveillance as an adjunct screening tool in gastrointestinal oncology [54].

#### 4.4.5. Oral–Hepatic Axis and Metabolic Dysfunction

Beyond the intestine, oral dysbiosis has been implicated in the pathogenesis of NAFLD and metabolic dysfunction-associated steatotic liver disease (MASLD). The systematic review by Huang et al. provides evidence linking oral dysbiosis to fatty liver disease via the oral–gut–liver axis. Translocated microbial products activate Kupffer cells, cause mitochondrial dysfunction, increase oxidative stress, and disrupt lipid metabolism, thereby promoting steatosis and fibrogenesis [55]. Additionally, reduced abundance of nitrate-reducing oral commensals, such as *Neisseria* and *Rothia*, impairs the nitrate–nitrite–NO pathway, reducing NO bioavailability and worsening endothelial and metabolic dysfunction [43]. The combination of microbial endotoxaemia and decreased NO-mediated antioxidative defence accelerates hepatic lipid accumulation and fibrosis progression.

#### 4.4.6. Clinical and Translational Implications

From IBD to colorectal and hepatic disease, converging evidence supports oral dysbiosis as both a biomarker and a modifiable risk factor in gastrointestinal pathology [14,43,50,51,53,54]. Integrating salivary microbial profiling with stool metagenomics may substantially improve early detection of cancer and metabolic disease. Periodontal therapy and optimising oral hygiene may help modulate systemic inflammation, gut permeability, and hepatic steatosis, potentially through the restoration of microbial eubiosis.

### 4.5. Endocrine and Metabolic System

#### 4.5.1. Overview of the Oral–Endocrine Axis

The endocrine system exemplifies bidirectional communication between oral microbial ecology and host metabolic regulation. Increasing evidence indicates that periodontitis and T2DM influence each other through interconnected immunometabolic mechanisms. Chronic hyperglycaemia increases gingival inflammation and promotes dysbiosis, while periodontal infection sustains systemic insulin resistance and worsens glycaemic control [7,56]. Unlike other systemic links discussed in this review, the oral–endocrine axis is characterised by disruptions in insulin signalling, metabolic endotoxaemia, and microbial metabolite activity, which connect oral biofilm dynamics with hepatic and adipose tissue physiology [7,10]. Beyond T2DM, oral microbial imbalance has been associated with gestational diabetes mellitus (GDM), characterised by dysbiotic shifts involving *Prevotella* and *Porphyromonas* species, increased systemic inflammatory cytokines, and impaired nitrate–nitrite–NO metabolism, which together contribute to adverse pregnancy outcomes (APOs) [43,67,68].

#### 4.5.2. Periodontitis and T2D: Functional Microbial Reprogramming

Functional metagenomic profiling has shown that diabetic status is associated with specific ecological and metabolic reprogramming within subgingival biofilms. Favale et al. identified a diabetes-linked functional signature enriched for LPS and peptidoglycan biosynthesis, as well as shifts in amino acid and fatty acid metabolism, consistent with a pro-inflammatory and oxidative phenotype [56]. Rather than implicating a single pathogen, these findings support a polymicrobial dysbiosis model, in which cumulative functional changes amplify insulin resistance through cytokine and oxidative pathways [7,56].

Clinically, individuals with T2DM exhibit greater periodontal destruction and higher systemic inflammatory markers, while hyperglycaemia alters crevicular nutrient gradients and innate defences, favouring the persistence of proteolytic anaerobes such as *Prevotella*, *Fusobacterium*, and *Porphyromonas* [7,56]. Notably, meta-analyses have shown that non-surgical periodontal therapy reduces HbA1c by approximately 0.4–0.6% at 3–6 months, a magnitude comparable to certain pharmacological interventions, supporting the therapeutic relevance of periodontal control in metabolic disease management [16]. These findings reinforce that periodontitis is both a complication of, and a modifiable risk factor for, diabetes-related dysmetabolism [7,16,56]. However, these effect sizes vary considerably depending on baseline glycaemic control, periodontal disease severity, sample size, and study design, and some larger trials report smaller or non-significant changes [73].

#### 4.5.3. Immunometabolic Mechanisms Linking Oral Dysbiosis to Insulin Resistance

Several interdependent mechanisms underpin the connection between oral dysbiosis and systemic insulin resistance:

(1) Cytokine-mediated interference with insulin signalling. Activation of TLR-2/4 by bacterial virulence factors initiates NF-κB cascades and elevates IL-1β, IL-6, and TNF-α, which inhibit insulin receptor signalling via serine phosphorylation of insulin receptor substrate 1 (IRS-1) [7].

(2) Oxidative and nitrosative stress. Dysbiotic biofilms and activated leukocytes increase reactive oxygen and nitrogen species, while depletion of nitrate-reducing commensals disrupts the nitrate–nitrite–NO pathway, reducing antioxidant capacity and insulin sensitivity [43].

(3) Metabolic endotoxaemia. Periodontal-derived LPS and microbe-associated molecular patterns translocate into the bloodstream, perpetuating low-grade systemic inflammation and hepatic insulin resistance [7,61].

(4) Microbial metabolite signalling. Altered SCFA and amino acid fluxes affect adipose and hepatic metabolism, contributing to dyslipidaemia and impaired glucose uptake [7,56].

(5) Advanced glycation end-product (AGE)—receptor for advanced glycation end-products (RAGE) synergy. Hyperglycaemia enhances AGE formation, while periodontal inflammation upregulates RAGE, creating a feed-forward loop of oxidative stress and inflammatory amplification [7].

#### 4.5.4. GDM and APOs

Pregnancy-induced hormonal and immune fluctuations make the periodontium particularly susceptible to inflammation and dysbiosis. Systematic reviews and meta-analyses show significant associations between maternal periodontitis, GDM, and APOs such as preterm birth, low birth weight, and pre-eclampsia [20,21]. Periodontal therapy during pregnancy has been shown to reduce systemic inflammatory markers (e.g., CRP, IL-6) and may improve obstetric outcomes [20,21]. Although causality cannot yet be firmly established, plausible mechanisms include endotoxin-induced placental cytokine activation, systemic oxidative stress, and increased maternal insulin resistance. These findings support the inclusion of oral health assessment in antenatal metabolic screening programmes [20,21].

#### 4.5.5. The Nitrate–Nitrite–NO Pathway in Endocrine Homeostasis

A complementary pathway linking oral microbiota to endocrine health involves the nitrate-reducing microbial community. Commensal genera such as *Neisseria* and *Rothia* convert dietary nitrate (NO_3_^−^) into nitrite (NO_2_^−^), which subsequently contributes to systemic NO production, a key modulator of vascular tone, mitochondrial efficiency, and insulin sensitivity [43]. Dysbiosis or excessive antiseptic use reduces these taxa, decreasing NO bioavailability and impairing endothelial and metabolic function [43]. This pathway, while intersecting with vascular mechanisms discussed in Section 4.2, also extends into endocrine regulation by modulating insulin sensitivity and NO-mediated metabolic signalling, illustrating the multidimensional metabolic impact of oral microbial eubiosis [42,43]. However, existing interventional evidence remains heterogeneous, with clinical benefits most evident when periodontal inflammation is reduced early and consistently maintained throughout follow-up [16].

#### 4.5.6. Salivary and Subgingival Biomarkers of Metabolic Risk

The oral cavity serves as a convenient diagnostic interface for metabolic disease surveillance. Salivary and subgingival samples reflect systemic inflammation and oxidative stress through detectable cytokines, microbial DNA, and metabolic by-products [20]. Elevated IL-6 and TNF-α concentrations, together with the functional microbial signatures described by Favale et al., have potential as biomarkers of metabolic deterioration and treatment response [20,56]. Integrating these indices with HbA1c and other clinical parameters could enable risk-adapted precision management in endocrine disorders [16,20,56]. Future translational application will depend on standardised saliva collection protocols, flow stimulation, and analytical calibration to ensure reproducibility across studies [20].

### 4.6. Renal System

#### 4.6.1. The Oral–Renal Axis: Conceptual Framework

Within the expanding field of systemic microbiome interactions, the oral–renal axis has emerged as a distinct pathway linking chronic inflammation, microbial dysbiosis, and metabolic imbalance. The kidneys, with their dense microvascular network and immunoregulatory function, are highly responsive to systemic inflammatory and oxidative signals originating from oral infection. Both periodontitis and CKD exhibit overlapping biological disturbances, persistent cytokine elevation, oxidative stress, and endothelial dysfunction, suggesting that oral microbial imbalance can aggravate renal injury, while renal impairment, in turn, disrupts the oral ecosystem [1,3,7,60].

#### 4.6.2. Periodontitis and CKD: A Bidirectional Relationship

Epidemiological evidence indicates that individuals with CKD are more susceptible to severe periodontal disease, while poor periodontal health accelerates renal function decline. These associations are not merely correlative but are supported by biological mechanisms involving systemic inflammation and metabolic endotoxaemia [59]. Interpretation of associations between periodontitis and renal outcomes is further complicated by shared risk factors, such as diabetes, hypertension, smoking, and socioeconomic status, which independently influence both periodontal and kidney health and may serve as important confounders in observational and short-term longitudinal studies. However, although consistent associative data exist, well-designed longitudinal human studies confirming bidirectional causality between oral dysbiosis and renal decline remain limited, highlighting the need for integrated oral–gut–renal microbiome analyses [59,60].

Randall et al. [60] demonstrated in experimental kidney injury models that gut dysbiosis, characterised by the loss of SCFA producers and enrichment of facultative pathogens, exhibits microbial features conceptually similar to those described in human periodontal dysbiosis, supporting an indirect, microbiome-mediated connection between the two systems. Pro-inflammatory cytokines released from periodontal tissues, such as IL-1β, IL-6, and TNF-α, circulate systemically and amplify glomerular inflammation and endothelial injury [7,59]. Meanwhile, bacterial fragments and endotoxins entering the bloodstream trigger TLR activation in renal tissue, promoting oxidative stress and fibrosis.

#### 4.6.3. Pathophysiological Pathways of the Oral–Renal Interaction

The interplay between oral dysbiosis and renal impairment occurs through several interconnected biological mechanisms:

(1) Cytokine overflow and immune activation. Periodontal inflammation leads to continuous release of pro-inflammatory mediators, increasing systemic immune activity and aggravating renal endothelial permeability [7,59].

(2) Oxidative imbalance and vascular injury. Persistent oral inflammation raises concentrations of reactive oxygen and nitrogen species, impairs renal microcirculatory function, and promotes tubular hypoxia [59,60].

(3) Microbial translocation and metabolic endotoxaemia. Circulating LPS and peptidoglycan fragments from oral bacteria stimulate renal macrophages and mesangial cells, activating MAPK and transforming growth factor beta (TGF-β) pathways, which promote fibrotic changes [59].

(4) Uraemia-associated oral dysbiosis. The accumulation of uraemic toxins in advanced CKD alters salivary composition and pH, suppressing protective commensals such as *Rothia* and *Neisseria*, while encouraging proteolytic anaerobes including *Porphyromonas* and *Prevotella* [59].

#### 4.6.4. Salivary Biomarkers in Renal Disease Monitoring and Preventive Implications

Saliva provides an accessible matrix for non-invasive detection of renal dysfunction. Increased salivary concentrations of urea, creatinine, uric acid, and inflammatory mediators have been observed in patients with declining renal function [20]. Similarly, oxidative stress indicators such as malondialdehyde (MDA) and nitrite show inverse correlations with glomerular filtration rate, reflecting systemic redox imbalance [23,60]. Combined assessment of salivary metabolites and microbial DNA profiles could therefore serve as an early diagnostic panel for CKD, linking oral diagnostics with nephrological surveillance [20]. Although these salivary biomarkers show promise, their clinical validity and utility for routine diagnosis of CKD remain under investigation and have not yet been fully established.

Integrated management, including dental, nutritional, and nephrological collaboration, could reduce systemic inflammation and slow renal decline [7,59,60]. Experimental probiotics and prebiotics designed to restore the microbiota along the oral–gut–renal continuum are currently under investigation and may represent next-generation adjuncts to conventional CKD therapy [6,60].

### 4.7. Reproductive Health and APOs

#### 4.7.1. Oral–Placental Axis and Systemic Inflammation

Pregnancy represents a unique immunometabolic equilibrium, characterised by profound endocrine, vascular, and microbial adaptations. These physiological changes increase the susceptibility of oral tissues to inflammation and dysbiosis. Periodontal inflammation during gestation elevates systemic concentrations of IL-6, TNF-α, and CRP, contributing to endothelial dysfunction and oxidative stress in the placenta [57,58,68]. The European Federation of Periodontology (EFP) and American Academy of Periodontology (AAP) Consensus Report by Sanz and Kornman established a biologically plausible framework linking periodontal infection to obstetric complications [57]. It proposed that the maternal oral microbiome acts as a peripheral source of inflammatory mediators capable of modulating placental vascular function. LPS and virulence factors from *P. gingivalis* and *F. nucleatum* can translocate into the placental microcirculation, where they activate TLR4 signalling in trophoblasts and endothelial cells, increasing cytokine production, endothelial activation, and microvascular permeability [57,58,68]. These events foster a pro-inflammatory intrauterine environment, increasing the risk of preterm birth, low birth weight, and pre-eclampsia [57,58,68].

#### 4.7.2. Maternal Microbiome and Immune Crosstalk

Beyond direct bacterial dissemination, oral dysbiosis affects pregnancy outcomes through immunological and metabolic reprogramming. SCFAs, such as butyrate, regulate trophoblastic cytokine tolerance and T-regulatory (Treg) cell differentiation. A reduced abundance of SCFA-producing commensals, together with increased *Prevotella* and *Selenomonas*, impairs immune tolerance and increases systemic inflammatory tone [57,58,68]. Salivary oxidative markers, including nitrite, MDA, and 8-hydroxy-2-deoxyguanosine, correlate with systemic redox imbalance, suggesting their potential as non-invasive indicators of maternal oxidative stress and immune activation. The oral cavity thus serves as a diagnostic proxy for overall inflammatory status during pregnancy.

#### 4.7.3. Molecular Mediators and Placental Crosstalk

Molecular evidence shows that microbial metabolites and cytokines of oral origin can alter placental gene expression profiles related to angiogenesis, oxidative balance, and nutrient transport [57,58,68]. Adhesins such as *F. nucleatum* FadA disrupt endothelial junctions and allow limited bacterial migration, while *P. gingivalis* gingipains up-regulate matrix metalloproteinases (MMPs) and impair trophoblast invasion [57,58,68]. The recent meta-analysis by Le et al. [58] confirmed a significant association between maternal periodontitis and pre-eclampsia, highlighting the mechanistic involvement of vascular remodelling and endothelial dysfunction in this relationship. These findings suggest that chronic oral inflammation may disrupt placental homeostasis by altering trophoblast signalling and vascular integrity.

#### 4.7.4. Immunometabolic Integration and Maternal Outcomes

The convergence of inflammatory, metabolic, and microbial pathways defines the complex immunometabolic landscape of pregnancy. Dysregulated NO bioavailability, caused by depletion of nitrate-reducing commensals such as *Neisseria* and *Rothia*, exacerbates endothelial dysfunction and reduces uteroplacental perfusion [6,43,57]. Simultaneously, cytokine spillover from periodontal inflammation may induce endothelial activation, ROS-mediated oxidative stress, and trophoblastic apoptosis, together precipitating placental ischaemia and reduced uteroplacental perfusion [57,58,68]. Therefore, maintaining oral eubiosis supports vascular stability and hormonal balance, whereas persistent periodontitis acts as a systemic metabolic stressor, compromising foetal development.

### 4.8. Autoimmune Diseases

#### 4.8.1. Periodontitis and RA: Shared Pathogenic Pathways

Periodontitis and RA share overlapping inflammatory mechanisms, including persistent cytokine activation, bone resorption, and impaired immune tolerance [17]. *P. gingivalis* produces PPAD, the only known bacterial enzyme that citrullinates arginine residues in host proteins, generating neoantigens that elicit anti-citrullinated protein antibodies (ACPAs), a diagnostic hallmark of RA [17]. *A. actinomycetemcomitans* enhances autoimmune activation by inducing NET formation through leukotoxin A, resulting in the release of citrullinated histones and the perpetuation of systemic inflammation [17,62]. Recent large-scale data integration by Gao et al. revealed that oral dysbiosis involving *P. gingivalis* correlates with enriched pathways for arginine and proline metabolism, citrullination, and LPS biosynthesis, which are strongly associated with systemic autoimmune gene networks [62]. These findings suggest that chronic periodontal infection may act as an environmental trigger for the initiation and exacerbation of RA in genetically predisposed individuals.

Several interventional studies and RCTs have examined the impact of non-surgical periodontal therapy on RA, reporting modest short-term improvements in disease activity scores (e.g., Disease Activity Score using 28 joint counts, DAS28). However, effects on autoantibody levels, including ACPA titres, have been inconsistent or absent, and the overall evidence remains limited by small sample sizes and short follow-up periods [74].

#### 4.8.2. Immunological and Molecular Intersections

Systemic cytokines elevated in both diseases (TNF-α, IL-1β, IL-6) enhance receptor activator of nuclear factor κB ligand (RANKL)-mediated osteoclastogenesis and promote bone destruction [17]. Autoantibodies and immune complexes originating in RA may infiltrate periodontal tissues, sustaining chronic inflammation. *P. gingivalis* virulence factors engage TLR2 and TLR4, activating NF-κB and p38-MAPK signalling, further intensifying the osteoimmune loop [6,17]. Multi-omic network analyses by Gao et al. demonstrated enrichment of TLR, Th17 cell differentiation, and antigen-processing pathways in oral and systemic datasets from autoimmune cohorts, supporting a shared molecular foundation between periodontal dysbiosis and systemic autoimmunity [62].

#### 4.8.3. SjD and Oral Microbial Dysbiosis

SjD exemplifies mucosal autoimmunity driven by disruption of the epithelial barrier and oral microbial imbalance. Chronic lymphocytic infiltration of the salivary glands reduces salivary flow, alters pH, and selects for proteolytic taxa while depleting symbiotic *Streptococcus* and *Haemophilus* species [61]. Metagenomic data show enrichment of *Prevotella*, *Leptotrichia*, and *Veillonella*, organisms capable of producing pro-inflammatory metabolites that activate Th17 responses [61]. Gao et al. confirmed overlapping functional modules between SjD and RA, including enhanced LPS and amino acid metabolism, as well as increased host antigen presentation pathways, reinforcing the concept of a “salivary microbiome–autoimmune interface” in which microbial dysbiosis and immune hyperactivation perpetuate each other [62].

#### 4.8.4. Epigenetic and Post-Translational Modifications in Autoimmune Susceptibility

Microbial metabolites such as butyrate and propionate regulate histone acetylation and DNA methylation, influencing the transcriptional control of inflammatory genes [6]. Oxidative and nitrosative stress in chronic periodontitis promotes protein nitration, carbonylation, and abnormal citrullination, generating neoantigens that disrupt immune tolerance [17]. Bioinformatic analyses by Gao et al. linked these post-translational modifications to microbial metabolic networks, particularly the LPS, polyamine, and sulphur compound pathways, suggesting an epigenetic link between oral dysbiosis and systemic autoimmune regulation [62].

#### 4.8.5. Oral Microbiome as an Immunoregulatory Network

The oral microbiome functions as an immunoregulatory ecosystem that shapes systemic immune tone. Commensals such as *Streptococcus mitis* and *Rothia mucilaginosa* promote Treg differentiation and IL-10 secretion, maintaining mucosal tolerance, while dysbiotic taxa such as *P. intermedia* and *F. nucleatum* promote Th17 polarisation and epithelial barrier damage [6]. Cross-disease meta-analyses indicate that conserved pro-inflammatory microbial clusters, particularly *Prevotella* and *Fusobacterium* species, are present across RA, SjD, and lupus cohorts, suggesting shared microbial contributors to systemic autoimmunity [62].

### 4.9. Oncological Aspects

#### 4.9.1. Oral Microbiota and Oral Squamous Cell Carcinoma (OSCC)

The oral microbiome plays an increasingly recognised role in oral carcinogenesis through chronic inflammation, oxidative stress, and genotoxic metabolite activity. The meta-analyses by Peter et al. [18] show that *F. nucleatum*, *P. gingivalis*, and *P. intermedia* are frequently enriched in OSCC tissue and saliva compared with healthy controls. These pathogens activate the NF-κB, β-catenin, and STAT3 pathways, promoting epithelial proliferation, angiogenesis, and resistance to apoptosis [18,65]. *P. gingivalis* also secretes gingipains, which degrade E-cadherin, facilitating EMT and invasion. *F. nucleatum* stimulates tumour progression through its adhesin FadA, which binds E-cadherin and enhances β-catenin nuclear translocation, leading to upregulation of the oncogenes cellular myelocytomatosis oncogene (c-Myc) and Cyclin D1 [18]. Microbial metabolism increases mutagenic pressure through acetaldehyde generated from ethanol oxidation and nitrosamines produced by nitrate reduction, both of which induce DNA strand breaks, methylation alterations, and impaired DNA repair. These effects are exacerbated by tobacco and alcohol use [8,18]. Across cohorts, these taxa show remarkable reproducibility, supporting their use as potential microbial biomarkers for early OSCC detection and prognostic monitoring [18,65].

#### 4.9.2. Molecular Mechanisms of Oral Carcinogenesis

Microbiota-induced carcinogenesis occurs through overlapping inflammatory and metabolic mechanisms. Chronic dysbiosis sustains the production of reactive oxygen and nitrogen species, leading to oxidative DNA lesions and chromosomal instability. Pro-inflammatory cytokines such as IL-6, IL-8, and TNF-α activate Janus kinase (JAK)/STAT and extracellular signal-regulated kinase (ERK) signalling, promoting continuous epithelial turnover and accumulation of mutations [18]. Microbial extracellular vesicles deliver microRNAs and virulence factors that reprogramme host gene expression, altering apoptosis, cell cycle checkpoints, and immune surveillance. In the tumour microenvironment, *F. nucleatum* suppresses T-cell cytotoxicity through its Fap2– T cell immunoreceptor with Ig and ITIM domains (TIGIT) interaction, while *P. gingivalis* promotes immune evasion by increasing programmed death ligand 1 (PD-L1) expression on epithelial cells [18,65]. These findings illustrate a multilayered oral–immune–tumour axis, in which local infection influences carcinogenic signalling, immune tolerance, and stromal activation.

#### 4.9.3. Microbiome–Tumour Microenvironment Interactions

Beyond carcinogenesis, the oral microbiome modulates tumour biology at multiple systemic sites. Bacterial components such as LPS and flagellin activate TLRs in tumour stromal and immune cells, promoting angiogenesis and fibroblast activation [18,65]. ROS generated by microbial metabolism enhance hypoxia-inducible factor-1α (HIF-1α) signalling, facilitating metabolic reprogramming towards glycolysis and promoting tumour survival in hypoxic niches. *F. nucleatum*-derived Fap2 also suppresses natural killer (NK) cell cytotoxicity, enabling immune evasion [18,65]. These immune–metabolic interactions integrate oral dysbiosis into the broader concept of the microbiome–tumour microenvironment, suggesting that microbial composition may influence therapeutic response and metastatic behaviour.

#### 4.9.4. Translational Oncology and Diagnostic Innovation

Salivary microbiome analysis provides a promising non-invasive method for early detection of oral and systemic cancers. Studies combining microbial and metabolomic profiling have identified predictive signatures involving *Fusobacterium*, *Porphyromonas*, and *Peptostreptococcus* species, achieving high diagnostic sensitivity for OSCC and CRC [14,51,52,54]. Liquid biopsy approaches that integrate microbial DNA with inflammatory cytokine panels could enable personalised risk stratification. Additionally, monitoring salivary microbial shifts may help assess treatment response and recurrence risk.

Although *F. nucleatum* and *P. gingivalis* are consistently reported as enriched in OSCC and CRC, most evidence to date comes from cross-sectional case–control studies, including analyses of tumour tissue, saliva, and faecal samples, as summarised in recent systematic reviews and meta-analyses [53]. Prospective cohort studies demonstrating temporal associations before cancer onset remain scarce. Consequently, while these taxa are promising mechanistic contributors and candidate biomarkers, their predictive value for cancer risk assessment requires validation in longitudinal studies.

### 4.10. Limitations

A limitation of this review is the exclusion of animal and in vitro studies, which may provide mechanistic insights. However, this approach was chosen to prioritise clinically relevant evidence. Additionally, the oral microbiome is not static throughout the lifespan. In childhood, microbial communities are highly dynamic and influenced by dentition stage, dietary transitions, and immune development. In contrast, ageing is associated with reduced microbial diversity, increased prevalence of pathogenic taxa, and greater susceptibility to dysbiosis, often exacerbated by systemic disease, medication use, and reduced salivary flow. Despite these recognised differences, age-specific evidence remains under-represented in systematic reviews and meta-analyses, limiting the ability to draw firm conclusions for specific populations.

## 5. Conclusions

The oral microbiome is increasingly recognised as a key determinant of systemic health, functioning as a dynamic and metabolically active ecosystem that extends beyond its anatomical boundaries. Evidence from multiple organ systems suggests that oral dysbiosis may be associated with systemic inflammation, oxidative stress, and metabolic changes outside the oral cavity.

Data from the neurological, cardiovascular, respiratory, GI, endocrine, renal, reproductive, autoimmune, and oncological fields reveal a unifying mechanistic triad: inflammation, oxidative stress, and metabolic disturbance. Within this systemic network, distinct yet overlapping “oral–systemic axes” emerge. The oral–gut axis mediates metabolic endotoxaemia and barrier disruption; the oral–vascular axis drives endothelial dysfunction and atherosclerosis; the oral–brain axis contributes to neuroinflammation and cognitive decline; and the oral–placental axis links periodontal infection to APOs. The oral microbiome may thus reflect and potentially influence systemic health, with possible associations to disease onset, progression, and recovery across multiple physiological systems.

Clinically, these findings suggest that oral health may play an important role in preventive medicine. Periodontal therapy has been shown to reduce systemic inflammation, improve glycaemic control, and positively influence cardiovascular, renal, and obstetric outcomes. Maintaining oral microbial eubiosis through consistent hygiene, probiotic and nitrate-based strategies, and modification of diet and lifestyle is a feasible, cost-effective adjunct to chronic disease prevention. Including oral health parameters in systemic disease screening protocols may therefore provide substantial benefits for early risk identification and therapeutic stratification.

At the translational level, advances in multi-omic technologies and artificial intelligence (AI)-assisted analytics promise to reveal causal links between microbial function and host response. Integration of metagenomic, metabolomic, transcriptomic, and immunological data will enable precise mapping of microbial networks that drive systemic pathology. Salivary diagnostics, due to their accessibility, reproducibility, and molecular depth, may soon serve as frontline tools for monitoring inflammatory status, metabolic risk, and treatment efficacy.

From a public health perspective, recognising the oral microbiome as a determinant of systemic disease positions dentistry as an integral part of holistic healthcare. The bidirectional relationship between oral ecology and general health highlights the need for interdisciplinary collaboration among dental medicine, internal medicine, and molecular biology. Incorporating microbial surveillance into precision health initiatives could transform population screening and chronic disease management by enabling early, non-invasive, and cost-effective monitoring of systemic inflammation.

By integrating molecular microbiology with clinical and translational science, the oral microbiome is no longer a peripheral curiosity but is now recognised as the missing link in the continuum of systemic health. Although mechanistic and clinical correlations are increasingly evident, causal relationships have yet to be fully clarified through longitudinal and interventional research. Future systematic reviews with standardised certainty-of-evidence assessments are needed to better quantify the strength of associations between oral dysbiosis and systemic health outcomes.

## Figures and Tables

**Table 1:** System-specific synthesis of high-level reviews (2019–2025).

System/Disease Area	Representative Systematic Reviews/Meta-Analyses	Type of Review (n of Primary Studies)	Core Findings	Translational/Clinical Significance
Neurological disorders (Alzheimer’s, Parkinson’s, cognitive function)	Maitre et al. [24]; Chaple-Gil et al. [28]; Mao et al. [29]	Meta-analyses (17–28 studies)	*P. gingivalis* gingipains induce microglial activation, tau hyperphosphorylation, and β-amyloid aggregation; pooled data show that oral dysbiosis is associated with an increased risk of dementia (OR ~1.7, 95% CI 1.3–2.2).	Supports the use of the salivary microbiome as a non-invasive biomarker for early neurodegeneration.
Cardiovascular diseases (atherosclerosis, hypertension, endothelial dysfunction)	Ye et al. [38]; Pizziolo et al. [39]; Murugesan & Al Khodor [42]	Umbrella & meta-analyses (~30 studies)	Periodontal therapy reduces CRP by 1–3 mg/L; loss of *Neisseria* or *Rothia* lowers NO bioavailability; periodontitis is associated with increased CVD risk (RR ~1.6, 95% CI 1.2–2.0).	Reinforces the importance of oral nitrate-reducing commensals for vascular homeostasis and supports oral care in the prevention of CVD.
Respiratory diseases (pneumonia, COPD, COVID-19)	Molina et al. [13]; Whiteside et al. [19]; Khadka et al. [48]; Kou et al. [49]	Systematic reviews (18–26 studies)	Structured oral care programmes reduce pneumonia by ~40% (RR 0.60, 95% CI 0.45–0.80); shared *Prevotella*/*Veillonella* signatures are observed in COPD and COVID-19.	Supports integrating oral hygiene into respiratory rehabilitation for frail adults.
Gastrointestinal/hepatic disorders (IBD, CRC, NAFLD)	Camañes-Gonzalvo et al. [14]; Zwezerijnen-Jiwa et al. [51]; Huang et al. [55]	Meta-analyses & systematic reviews (25–40 studies)	*F. nucleatum* and *P. gingivalis* are enriched in CRC (OR ~2.2, 95% CI 1.5–3.0); oral dysbiosis increases Kupffer cell activation and steatosis.	Supports combined salivary and faecal microbial panels for early screening of CRC and NAFLD.
Endocrine/reproductive systems (T2DM, GDM, pre-eclampsia)	Favale et al. [56]; Le et al. [58]	Umbrella + meta-analyses (≥40 studies)	Bidirectional association between periodontitis–T2DM (OR ~1.6, 95% CI 1.3–2.0); periodontal therapy reduces HbA1c by 0.4–0.6%; maternal periodontitis is associated with an increased risk of pre-eclampsia (RR ~1.7).	Supports routine periodontal assessment in metabolic and antenatal care.
Renal disease (CKD)	Baciu et al. [59]; Randall et al. [60]	Systematic reviews (12–18 studies)	Periodontitis is associated with an increased risk of CKD (OR ~1.5, 95% CI 1.1–2.0); cytokine overflow and oxidative stress exacerbate glomerular injury.	Suggests salivary creatinine and oxidative markers as early indicators of CKD.
Autoimmune disorders (RA, SjD)	Dolcezza et al. [17]; Mieliauskaitė & Kontenis [61]; Gao et al. [62]	Integrative multi-omics reviews (>20 studies)	*P. gingivalis* PPAD-citrullination and *A. actinomycetemcomitans* leukotoxin induce NETosis; shared Th17/TLR pathways.	Reinforces oral dysbiosis as a molecular trigger of RA and SjD.
Oncological disorders (OSCC, CRC)	Peter et al. [18]; Reitano et al. [54]	Meta-analyses (>30 studies)	*F. nucleatum* and *P. gingivalis* activate the NF-κB/β-catenin and ROS pathways, leading to → tumour progression (OR ~2.3, 95% CI 1.6–3.4).	Supports salivary microbial DNA profiling for early cancer risk assessment.

Abbreviations: OR, odds ratio; CI, confidence interval; CRP, C-reactive protein; NO, nitric oxide; CVD, cardiovascular diseases; COPD, chronic obstructive pulmonary disease; COVID-19, coronavirus disease 2019; IBD, inflammatory bowel disease; CRC, colorectal cancer; NAFLD, non-alcoholic fatty liver disease; T2DM, type 2 diabetes mellitus; GDM, gestational diabetes mellitus; CKD, chronic kidney disease; RA, rheumatoid arthritis; SjD, Sjögren’s disease; PPAD, Porphyromonas gingivalis peptidylarginine deiminase; NETosis, neutrophil extracellular trap formation; TLR, Toll-like receptor; OSCC, oral squamous cell carcinoma; NF-κB, nuclear factor kappa B; ROS, reactive oxygen species; DNA, deoxyribonucleic acid.

**Table 2:** Mechanistic and quantitative integration of systemic pathways linking oral dysbiosis to multiple organ outcomes.

Mechanistic Axis	Representative Quantitative Signals (Meta-Analytic Data)	Core Biological Mediators/Pathways	Principal Systemic Outcomes	Therapeutic/Diagnostic Translation
Inflammatory amplification	IL-6 ↑ 25–35%; CRP ↑ 2–3 mg/L; periodontal therapy → IL-6 ↓ ≈ 30% [16,29,30,38,39,40,42,43,56]	TLR2/4 → NF-κB → IL-1β/IL-6/TNF-α; NLRP3 inflammasome	Endothelial activation, insulin resistance, neuroinflammation [20,22,23,24,25,26,27,28,29,30,31,38,39,40]	Cytokine-attenuating periodontal therapy; salivary IL-6 and CRP as inflammatory biomarkers [20,29,38,42,56,63]
Oxidative/nitrosative stress	MDA ↑ 35–45%; NO ↓ ≈ 25% in dysbiosis [30,34,43,50,55,64]	Mitochondrial dysfunction; NADPH oxidase activation; eNOS uncoupling	Vascular stiffness, hepatic steatosis, cognitive decline [24,34,43,55]	Antioxidant and NO-restoring interventions; dietary nitrate supplementation [30,42,43,64]
Metabolic endotoxaemia	Circulating LPS ↑ 2–3× versus controls [14,50,51,52,53,54,55,56]	Endothelial and hepatic TLR4 activation; Kupffer cell cytokinaemia	NAFLD, insulin resistance, systemic low-grade inflammation [14,50,51,52,53,54,55,56]	Targeted prebiotics and probiotics to reduce LPS translocation [6,50,55]
Immune mimicry/maladaptation	ACPAs ↑ ≈ 1.8× in RA with periodontitis [17,61,62]	*P. gingivalis* PPAD-mediated citrullination; *A. actinomycetemcomitans* leukotoxin-induced NETosis; Th17 cell expansion	Autoimmune flare, bone resorption, connective tissue damage [17,61,62]	Proof-of-concept immunomodulatory microbiome therapies [17,62]
Oncogenic transformation	*F. nucleatum* enrichment OR ≈ 2.0–2.3 (95% CI 1.6–3.4); oxidative DNA lesions ↑ 30–40% [14,18,51,52,53,54,65]	NF-κB/STAT3/β-catenin activation; EMT; PD-L1 up-regulation	OSCC and CRC progression [14,18,51,52,53,54,65,66]	Microbiome-informed cancer screening and adjunctive monitoring [51,52,53,54]
Nitrate-reduction/NO-pathway depletion	Nitrate reductase activity ↓ > 50% after antiseptic use; loss of *Neisseria* and *Rothia* [42,43]	Reduced nitrate → nitrite → NO conversion; ADMA-mediated NOS inhibition [43]	Hypertension, placental hypoperfusion, and endothelial dysfunction [43,67,68]	Limit use of bactericidal mouthwash; preserve nitrate-reducing commensals; promote dietary nitrate [42,43]

Abbreviations: IL-6, interleukin 6; CRP, C-reactive protein; TLR, Toll-like receptor; NF-κB, nuclear factor kappa B; IL-1β, interleukin-1 beta; TNF-α, tumour necrosis factor alpha; NLRP3, NOD-like receptor pyrin domain-containing 3; MDA, malondialdehyde; NO, nitric oxide; NADPH, nicotinamide adenine dinucleotide phosphate; eNOS, endothelial nitric oxide synthase; LPS, lipopolysaccharide; NAFLD, non-alcoholic fatty liver disease; ACPAs, anti-citrullinated protein antibodies; RA, rheumatoid arthritis; PPAD, Porphyromonas gingivalis peptidylarginine deiminase; NETosis, neutrophil extracellular trap; OR, odds ratio; CI, confidence interval; DNA, deoxyribonucleic acid; STAT3, signal transducer and activator of transcription 3; EMT, epithelial–mesenchymal transition; PD-L1, programmed death ligand 1; OSCC, oral squamous cell carcinoma; CRC, colorectal cancer; ADMA, asymmetric dimethylarginine; NOS, nitric oxide synthase.

## Data Availability

No new data were created or analyzed in this study. Data sharing is not applicable to this article.

## Supplementary Materials

The following supporting information can be downloaded at: https://www.mdpi.com/article/10.3390/life16020294/s1, Table S1: SANRA Checklist.

## Abbreviations

The following abbreviations are used in this manuscript:

MEDLINE	Medical Literature Analysis and Retrieval System Online
CVD	Cardiovascular disease
T2DM	Type 2 diabetes mellitus
CKD	Chronic kidney disease
PAMPs	Pathogen-associated molecular patterns
TLRs	Toll-like receptors
IL-1β	Interleukin 1 beta
IL-6	Interleukin 6
TNF-α	Tumour necrosis factor alpha
SCFAs	Short-chain fatty acids
SANRA	Scale for the Assessment of Narrative Review Articles
MeSH	Medical Subject Headings
COVID-19	Coronavirus Disease 2019
GI	Gastrointestinal
PRISMA	Preferred Reporting Items for Systematic Reviews and Meta-Analyses
NF-κB	Nuclear Factor kappa B
NLRP3	NOD-like receptor pyrin domain-containing 3
ROS/RNS	Reactive Oxygen Species/Reactive Nitrogen Species
NO	Nitric oxide
OR	Odds ratio
CI	Confidence Interval
MAPK	Mitogen-Activated Protein Kinase
LPS	Lipopolysaccharides
BBB	Blood–brain barrier
CRP	C-reactive protein
IL-10	Interleukin 10
CRC	Colorectal cancer
DNA	Deoxyribonucleic acid
NAFLD	Non-alcoholic fatty liver disease
HbA1c	Haemoglobin A1c/Glycated haemoglobin A1c
PPAD	*Porphyromonas gingivalis* peptidylarginine deiminase
NETosis	Neutrophil extracellular trap formation
RA	Rheumatoid arthritis
SjD	Sjögren’s disease
STAT3	Signal Transducer and Activator of Transcription 3
EMT	Epithelial–mesenchymal transition
AD	Alzheimer’s disease
CNS	Central nervous system
PD	Parkinson’s disease
HPA	Hypothalamic–pituitary–adrenal
BDNF	Brain-derived neurotrophic factor
NOD	Nucleotide-binding Oligomerization Domain
NLRs	NOD-like receptors
HSP	Heat shock protein
ICAM-1	Intercellular Adhesion Molecule-1
VCAM-1	Vascular Cell Adhesion Molecule-1
ADMA	Asymmetric dimethylarginine
NOS	Nitric oxide synthase
IE	Infective endocarditis
NGS	Next-generation sequencing
COPD	Chronic obstructive pulmonary disease
ACE2	Angiotensin-converting enzyme 2
IBD	Inflammatory bowel disease
Th17	T helper 17 cells
MASLD	Metabolic dysfunction-associated steatotic liver disease
GDM	Gestational diabetes mellitus
APOs	Adverse pregnancy outcomes
IRS-1	Insulin receptor substrate-1
AGE	Advanced glycation end-product
RAGE	Receptor for advanced glycation end-products
TGF-β	Transforming Growth Factor beta
MDA	Malondialdehyde
EFP	European Federation of Periodontology
AAP	American Academy of Periodontology
Treg	Regulatory T cells
FadA	Fusobacterium adhesin A
MMP	Matrix Metalloproteinases
ACPAs	Anti-citrullinated protein antibodies
RANKL	Receptor Activator of Nuclear Factor κB Ligand
OSCC	Oral Squamous Cell Carcinoma
c-Myc	Cellular Myelocytomatosis oncogene
JAK	Janus Kinase
ERK	Extracellular signal-Regulated Kinase
RNA	Ribonucleic acid
Fap2	Fusobacterium adhesin protein 2
TIGIT	T cell immunoreceptor with Ig and ITIM domains
ITIM	Immunoreceptor Tyrosine-based Inhibitory Motif
PD-L1	Programmed death ligand 1
HIF-1α	Hypoxia-inducible factor-1α
NK	Natural killer cell
AI	Artificial intelligence
GRADE	Grading of Recommendations Assessment, Development and Evaluation
SSR	Summary Risk Ratio
DAS28	Disease Activity Score using 28 joint counts
MR	Mendelian randomisation

## References

[1] Deo P.N., Deshmukh R. (2019). Oral microbiome: Unveiling the fundamentals. J. Oral Maxillofac. Pathol..

[2] Tian S., Ding T., Li H. (2024). Oral microbiome in human health and diseases. mLife.

[3] Lamont R.J., Koo H., Hajishengallis G. (2018). The oral microbiota: Dynamic communities and host interactions. Nat. Rev. Microbiol..

[4] Rajasekaran J.J., Krishnamurthy H.K., Bosco J., Jayaraman V., Krishna K., Wang T., Bei K. (2024). Oral microbiome: A review of Its Impact on Oral and Systemic Health. Microorganisms.

[5] Marsh P.D. (1994). Microbial ecology of dental plaque and its significance in health and disease. Adv. Dent. Res..

[6] Bostanghadiri N., Kouhzad M., Taki E., Elahi Z., Khoshbayan A., Navidifar T., Darban-Sarokhalil D. (2024). Oral microbiota and metabolites: Key players in oral health and disotder, and microbiota-based therapies. Front. Microbiol..

[7] Peng X., Cheng L., You Y., Tang C., Ren B., Li Y., Xu X., Zhou X. (2022). Oral microbiota in human systemic diseases. Int. J. Oral Sci..

[8] Senaratne N.L.M., On C.Y., Shetty N.Y., Gopinath D. (2024). Effect of different forms of tobacco on the oral microbiome in healthy adults: A systematic review. Front. Oral Health.

[9] Olsen I., Yamazaki K. (2019). Can oral bacteria affect the microbiome of the gut?. J. Oral Microbiol..

[10] Willis J.R., Gabaldón T. (2020). The Human Oral Microbiome in Health and Disease: From Sequences to Ecosystems. Microorganisms.

[11] Martins C.C., Lockhart P.B., Firmino R.T., Klimartin C., Cahill T.J., Dayer M., Occhi-Alexandre I.G.P., Lai H., Ge L., Thornhill M.H. (2024). Bacteremia following different oral procedures: Systematic review and meta-analysis. Oral Dis..

[12] Arbildo-Vega H.I., Cruzado-Oliva F.H., Coronel-Zubiate F.T., Meza-Málaga J.M., Luján-Valencia S.A., Luján-Urviola E., Echevarria-Goche A., Farje-Gallardo C.S., Castillo-Cornock T.B., Serquen-Olano K. (2024). Periodontal disease and cardiovascular disease: Umberlla review. BMC Oral Health.

[13] Molina A., Huck O., Herrera D., Montero E. (2023). The association between respiratory diseases and periodontitis: A systematic review and meta-analysis. J. Clin. Periodontol..

[14] Camañes-Gonzalvo S., Montiel-Company J.M., Lobo-de-Mena M., Safont-Aguilera M.J., Fernández-Diaz A., López-Roldán A., Paredes-Gallardo V., Bellot-Arcís C. (2024). Relationship between oral microbiota and colorectal cancer: A systematic review. J. Periodontal Res..

[15] Ikbal S.K.A., Yadav S.K., Mehrotra R., Fatima T., Sharda A., Gupta S. (2023). Oral Microbiota as a Diagnostic Biomarker of Digestive Cancer: A Systematic Review. J. Contemp. Dent. Pract..

[16] Umezaki Y., Yamashita A., Nishimura F., Naito T. (2025). The role of periodontal treatment on the reduction of hemoglobinA1c, comparing with existing medication therapy: A systematic review and meta-analysis. Front. Clin. Diabetes Healthc..

[17] Dolcezza S., Flores-Fraile J., Lobo-Galindo A.B., Montiel-Company J.M., Zubizarreta-Macho Á. (2024). Relationship Between Rheumatoid Arthritis and Periodontal Disease-Systematic Review and Meta-Analysis. J. Clin. Med..

[18] Peter T.K., Withanage M.H.H., Comnick C.L., Pandleton C., Dabdoub S., Ganesan S., Drake D., Banas J., Xie X.J., Zeng E. (2022). Systematic review and meta-analysis of oral squamous cell carcinoma associated oral microbiome. Front. Microbiol..

[19] Whiteside S.A., McGinniss J.E., Collman R.G. (2021). The lung microbiome: Progress and promise. J. Clin. Investig..

[20] Meleti M., Cassi D., Vescovi P., Setti G., Pertinhez T.-A., Pezzi M.-E. (2020). Salivary biomarkers for diagnosis of systemic diseases and malignant tumors. A systematic review. Med. Oral Patol. Oral Cir. Bucal..

[21] Jin D.-M., Morton J.T., Bonneau R. (2024). Meta-analysis of the human gut microbiome uncovers shared and distinct microbial signatures between diseases. mSystems.

[22] Liu S., Dashper S.G., Zhao R. (2023). Association Between Oral Bacteria and Alzheimer’s Disease: A Systematic Review and Meta-Analysis. J. Alzheimers Dis..

[23] Bello-Corral L., Alves-Gomes L., Fernández-Fernández J.A., Fernández-García D., Casado-Verdejo I., Sánchez-Valdeón L. (2023). Implications of gut and oral microbiota in neuroinflammatory responses in Alzheimer’s disease. Life Sci..

[24] Maitre Y., Mahalli R., Micheneau P., Delpierre A., Amador G., Denis F. (2021). Evidence and Therapeutic Perspectives in the Relationship between the Oral Microbiome and Alzheimer’s Disease: A Systematic Review. Int. J. Environ. Res. Public Health.

[25] Yussof A., Yoon P., Krkljes C., Schweinberg S., Cottrell J., Chu T., Chang S.L. (2020). A meta-analysis of the effect of binge drinking on the oral microbiome and its relation to Alzheimer’s disease. Sci. Rep..

[26] Murcia-Flores L., Sánchez-García A., Pecci-Lloret M.P., Rodríguez-Lozano F.J. (2025). Association between oral dysbiosis and Parkinson’s disease: A systematic review. Front. Cell Infect. Microbiol..

[27] Cao D., Yang J., He Y., Zheng X., Li Y., Chen Y., Tu Y. (2025). Altered oral microbiome composition in mental disorders: A systematic review and meta-analysis. J. Oral Microbiol..

[28] Chaple-Gil A.M., Santiesteban-Velázquez M., Urbizo Vélez J.J. (2025). Association Between Oral Microbiota Dysbiosis and the Risk of Dementia: A Systematic Review. Dent. J..

[29] Mao S., Huang C.-P., Lan H., Lau H.-G., Chiang C.-P., Chen Y.-W. (2022). Association of periodontitis and oral microbiomes with Alzheimer’s disease: A narrative systematic review. J. Dent. Sci..

[30] Karaduran K., Aydogdu A., Gelisin O., Gunpinar S. (2023). Investigating the potential clinical impact of periodontitis on the progression of Alzheimer’s disease: A prospective cohort study. Clin. Oral Investig..

[31] Jockusch J., Hopfenmüller W., Nitschke I. (2021). Influence of cognitive impairment and dementia on oral health and the utilization of dental services: Findings of the Oral Health, Bite force and Dementia Study (OrBiD). BMC Oral Health.

[32] Paudel D., Uehara O., Giri S., Yoshida K., Morikawa T., Kitagawa T., Matsuoka H., Miura H., Toyofuku A., Kuramitsu Y. (2022). Effect of psychological stress on the oral–gut microbiota and the potential oral-gut-brain axis. Jpn. Dent. Sci. Rev..

[33] Adil N.A., Omo-Erigbe C., Yadav H., Jain S. (2025). The Oral–Gut microbiome –Brain Axis in Cognition. Microorganisms.

[34] Clasen F., Yildirim S., Arıkan M., Garcia-Guevara F., Hanoğlu L., Yılmaz N.H., Şen A., Celik H.K., Neslihan A.A., Demir T.K. (2025). Microbiome signatures of virulence in the oral–gut–brain axis influence Parkinson’s disease and cognitive decline pathophysiology. Gut Microbes.

[35] García-Rios P., Pecci-Lloret M.R., Pecci-Lloret M.P., Murcia-Flores L., Pérez-Guzmán N. (2025). Association Between Oral Dysbiosis and Depression: A Systematic Review. J. Clin. Med..

[36] Wang M., Wang Z., Zhao D., Yu Y., Wei F. (2024). Periodontitis causally affects the brain cortical structure: A Mendelian randomization study. J. Periodontal. Res..

[37] Deng Z., Li J., Zhang Y., Zhang Y. (2024). No genetic causal associations between periodontitis and brain atrophy or cognitive impairment: Evidence from a comprehensive bidirectional Mendelian randomization study. BMC Oral Health.

[38] Ye Z., Cao Y., Miao C., Liu W., Dong L., Lv Z., Iheozor-Ejiofor Z., Li C. (2022). Periodontal therapy for primary or secondary prevention of cardiovascular disease in people with periodontitis. Cochrane Database Syst. Rev..

[39] Pizziolo P.G., Silva-Lovato C.H., Clemente L.M., Carandina A., Salgado H.C., Silva T.M.d., Tobaldini E., Montano N., Ribeiro A.B. (2025). The role of oral microbiota and tooth loss in cardiovascular disease risk: A systematic review. Biofouling.

[40] Akhi R., Lavrinienko A., Hakula M., Hindström R., Wang C., Nissinen A., Kullaa A.M., Salo T., Kaikkonen K., Tervonen T. (2025). Oral microbiota linking humoral response, periodontitis and atherosclerosis. J. Clin. Periodontol..

[41] Wu Y., Xing L., Lu L., Liu S., Zhao D., Lin L., Wang S., Li C., Pan Y. (2024). Alterations in the salivary microbiome and metabolism in patients with carotid atherosclerosis from rural Northeast China. J. Am. Heart Assoc..

[42] Murugesan S., Al Khodor S. (2023). Salivary microbiome and hypertension in the Qatari population. J. Transl. Med..

[43] Morou-Bermúdez E., Torres-Colón J.E., Bermúdez N.S., Patel R.P., Joshipura K.J. (2022). Pathways linking oral bacteria, nitric oxide metabolism, and health. J. Dent. Res..

[44] Gomes B.P.F.A., Berber V.B., Chiarelli-Neto V.M., Aveiro E., Chapola R.C., Passini M.R.Z., Lopes E.M., Chen T., Paster B.J. (2023). Microbiota present in combined endodontic-periodontal diseases and its risks for endocarditis. Clin. Oral Investig..

[45] Bondonno C.P., Liu A.H., Croft K.D., Considine M.J., Puddey I.B., Woodman R.J., Hodgson J.M. (2015). Antibacterial mouthwash blunts oral nitrate reduction and increases blood pressure in treated hypertensive men and women. Am. J. Hypertens..

[46] Tan L., Zhong M.-M., Liu Q., Chen Y., Zhao Y.-Q., Zhao J., Dusenge M.A., Feng Y., Ye Q., Hu J. (2023). Potential interaction between the oral microbiota and COVID-19: A meta-analysis and bioinformatics prediction. Front. Cell Infect. Microbiol..

[47] Ganesan S.M., Peter T.K., Withanage M.H.H., Boksa F., Zeng E., Martinez A., Dabdoub S.M., Dhingra K., Hernandez-Kapila Y. (2000). COVID-19 associated oral and oropharyngeal microbiome: Systematic review and meta-analysis. Periodontology.

[48] Khadka S., Khan S., King A., Goldberg L.R., Crocombe L., Bettiol S. (2021). Poor oral hygiene, oral microorganisms and aspiration pneumonia risk in older people in residential aged care: A systematic review. Age Ageing.

[49] Kou Z., Liu K., Qiao Z., Wang Y., Li Y., Li Y., Yu X., Han W. (2024). The alterations of oral, airway and intestine microbiota in chronic obstructive pulmonary disease: A systematic review and meta-analysis. Front. Immunol..

[50] Chen H., Peng L., Wang Z., He Y., Zhang X. (2024). Exploring the causal relationship between periodontitis and gut microbiome: Unveiling the oral–gut and gut–oral axes through bidirectional Mendelian randomization. J. Clin. Periodontol..

[51] Zwezerijnen-Jiwa F.H., Sivov H., Paizs P., Zafeiropoulou K., Kinross J. (2023). A systematic review of microbiome-derived biomarkers for early colorectal cancer detection. Neoplasia.

[52] Avuthu N., Guda C. (2022). Meta-Analysis of Altered Gut Microbiota Reveals Microbial and Metabolic Biomarkers for Colorectal Cancer. Microbiol. Spectr..

[53] Navarro-Sánchez A., Nieto-Vitoria M.Á., López-López J.A., Martínez-Crespo J.J., Navarro-Mateu F. (2025). Is the oral pathogen, Porphyromonas gingivalis, associated to colorectal cancer?: A systematic review. BMC Cancer.

[54] Reitano E., de’Angelis N., Gavriilidis P., Gaiani F., Memeo R., Inchingolo R., Bianchi G., de’Angelis G.L., Carra M.C. (2021). Oral Bacterial Microbiota in Digestive Cancer Patients: A Systematic Review. Microorganisms.

[55] Huang M., Zhang X., Zhou R., Song Y., Zhang J., Wu J. (2024). Advances in the study of oral microbiota and metabolism associated fatty liver disease: A systematic review. Front. Cell. Infect. Microbiol..

[56] Favale N., Farina R., Carrieri A., Simonelli A., Severi M., Sabbioni S., Trombelli L., Scapoli C. (2024). Functional profile of oral plaque microbiome: Further insight into the bidirectional relationship between type 2 diabetes and periodontitis. Mol. Oral Microbiol..

[57] Sanz M., Kornman K., working group 3 of the joint EFP/AAP workshop (2013). Periodontitis and adverse pregnancy outcomes: Consensus report of the Joint EFP/AAP Workshop on Periodontitis and Systemic Diseases. J. Periodontol..

[58] Le Q.-A., Akhter R., Coulton K.M., Vo N.T.N., Duong L.T.Y., Nong H.V., Yaacoub A., Condous G., Eberhard J., Nanan R. (2022). Periodontitis and Preeclampsia in Pregnancy: A Systematic Review and Meta-Analysis. Matern. Child Health J..

[59] Baciu S.F., Mesaroș A.-Ș., Kacso I.M. (2023). Chronic Kidney Disease and Periodontitis Interplay-A Narrative Review. Int. J. Environ. Res. Public Health.

[60] Randall D.W., Kieswich J., Hoyles L., McCafferty K., Curtis M., Yaqoob M.M. (2023). Gut Dysbiosis in Experimental Kidney Disease: A Meta-Analysis of Rodent Repository Data. J. Am. Soc. Nephrol..

[61] Mieliauskaitė D., Kontenis V. (2023). Insights into Microbiota in Sjögren’s Syndrome. Medicina.

[62] Gao L., Cheng Z., Zhu F., Bi C., Shi Q., Chen X. (2022). The Oral Microbiome and Its Role in Systemic Autoimmune Diseases: A Systematic Review of Big Data Analysis. Front. Big Data.

[63] Gupta V., Dawar A., Das S.K., Yadav V.S., Nalwa V., Haidrus R., Purohit B.M., Goyal L. (2025). Cardiovascular Biomarkers in Periodontitis: A Systematic Review and Meta-Analysis. Oral Dis..

[64] Rei N., Grunho M., Mendes J.J., Fonseca J. (2024). Microbiota orchestra in Parkinson’s disease: The nasal and oral maestros. Biomedicines.

[65] Ahmad S., Jayamanne D., Bergamin S., Lawless A., Guminski A., Lee A., Yuile A., Wheeler H., Eade T., Back M. (2025). Oral Microbiome as a Biomarker and Therapeutic Target in Head and Neck Cancer: Current Insights and Future Directions. Cancers.

[66] Zhang K., He C., Qiu Y., Li X., Hu J., Fu B. (2023). Association of oral microbiota and periodontal disease with lung cancer: A systematic review and meta-analysis. J. Evid. Based Dent. Pract..

[67] Salama M., Al-Taiar A., McKinney D.C., Rahman E., Merchant A.T. (2024). The impact of scaling and root planning combined with mouthwash during pregnancy on preterm birth and low birth weight: A systematic review and meta-analysis. BMC Pregnancy Childbirth.

[68] Machado V., Ferreira M., Lopes L., Mendes J.J., Botelho J. (2023). Adverse Pregnancy Outcomes and Maternal Periodontal Disease: An Overview on Meta-Analytic and Methodological Quality. J. Clin. Med..

[69] Barrio C., Arias-Sánchez S., Martín-Monzón I. (2022). The gut microbiota-brain axis, psychobiotics and its influence on brain and behaviour: A systematic review. Psychoneuroendocrinology.

[70] Wang B., Zhang C., Shi C., Zhai T., Zhu J., Wei D., Shen J., Liu Z., Jia K., Zhao L. (2024). Mechanisms of oral microflora in Parkinson’s disease. Behav. Brain Res..

[71] Dickson R.P., Erb-Downward J.R., Prescott H.C., Martinez F.J., Curtis J.L., Lama V.N., Huffnagle G.B. (2014). Analysis of culture-dependent versus culture-independent techniques for identification of bacteria in clinically obtained bronchoalveolar lavage fluid. J. Clin. Microbiol..

[72] Qi X., Northridge M.E., Hu M., Wu B. (2022). Oral health conditions and COVID-19: A systematic review and meta-analysis of the current evidence. Aging Health Res..

[73] Chen Y.F., Zhan Q., Wu C.Z., Yuan Y.H., Chen W., Yu F.Y., Li Y., Li L.J. (2021). Baseline HbA1c Level Influences the Effect of Periodontal Therapy on Glycemic Control in People with Type 2 Diabetes and Periodontitis: A Systematic Review on Randomized Controlled Trails. Diabetes Ther..

[74] Mustufvi Z., Twigg J., Kerry J., Chesterman J., Pavitt S., Tugnait A., Mankia K. (2022). Does periodontal treatment improve rheumatoid arthritis disease activity? A systematic review. Rheumatol. Adv. Pract..

