# Supplementary material for: The Oral Microbiome and Systemic Health: Current Insights into the Mouth–Body Connection
Source: Life (Basel). 2026 Feb 9;16(2):294. doi: 10.3390/life16020294 (PMC12941995; doi:10.3390/life16020294)
Supplement: Supplementary file 1 [file life-16-00294-s001.zip › life-4127899-supplementary.pdf]

Table S1: SANRA Checklist

Item	Score (0-2)	Justification
**Justification of the article's importance**	2	The introduction clearly explains the importance of the oral microbiota for systemic health and links it to various organ systems, highlighting the relevance of the topic.
**Statement of aims**	2	The objectives of the review are clearly defined and include an overview of mechanisms, biomarkers, and potential clinical implications.
**Literature search**	2	A comprehensive literature search was conducted using PubMed (MEDLINE), Web of Science, Scopus, and Cochrane databases. Eligibility and exclusion criteria are clearly defined, focusing on systematic reviews, umbrella reviews, and meta-analyses published between January 2019 and July 2025, with selective inclusion of older seminal studies for mechanistic insights. The search strategy is transparent and reproducible, although the inclusion of older studies was based on the authors' judgement.
**Referencing**	2	Appropriate, recent and relevant sources were used, and references were cited consistently and correctly.
**Scientific reasoning**	2	Analysis and synthesis of evidence are logical, highlighting the experimental or associative nature of the evidence as appropriate.
**Presentation of data**	2	The text is clearly structured, and the tables and figures are informative and aid understanding. The inclusion of a schematic representation (Figure 2) provides additional visual support.

Total Score: 12/12
